# Optimising 24-Hour movement behaviours in preschoolers through parenting practices: an evidence-based intervention study

**DOI:** 10.1186/s12966-025-01863-z

**Published:** 2025-12-12

**Authors:** Marga Decraene, Aaron Miatke, Dorothea Dumuid, Greet Cardon, Maïté Verloigne, Ruth De Bruyne, Vera Verbestel, Marieke De Craemer

**Affiliations:** 1 Department of Rehabilitation Sciences, Ghent University, Ghent, 9000 Belgium; 2 Department of Movement and Sports Sciences, Ghent University, Ghent, 9000 Belgium; 3 Allied Health and Human Performance, Alliance for Research in Exercise Nutrition and Activity (ARENA), University of South Australia, Adelaide, Australia; 4 Department of Public Health and Primary Care, Ghent University, Ghent, 9000 Belgium; 5 Department of Pediatric Gastroenterology, Hepatology and Nutrition, Ghent University, Ghent, Belgium; 6 Institute of Nutrition and Translational Research in Metabolism (NUTRIM), Department of Health Promotion, Faculty of Health, Medicine & Life Science, Maastricht University, Maastricht, The Netherlands; 7 Care and Public Health Research Institute (CAPHRI), Department of Health Promotion, Faculty of Health, Medicine & Life Science, Maastricht University, Maastricht, The Netherlands

**Keywords:** 24-hour movement behaviours, Parenting practices, Preschoolers, Physical activity, Sedentary behaviour, Screen time, Sleep, Intervention, Self-determination theory.

## Abstract

**Background:**

Establishing healthy 24-hour movement behaviours early in life is crucial for long-term health. However, few preschoolers comply with the 24-hour movement behaviour guidelines. Given the pivotal role of parents in shaping children’s health habits, interventions targeting parenting practices may optimise these behaviours in preschoolers. This study evaluates the effectiveness of the ‘Move ARound And Get Active’ (MARGA) intervention in improving preschoolers’ 24-hour movement behaviour composition, guideline compliance, and parents’ parenting practices.

**Methods:**

A two-armed, non-equivalent pretest-post-test control group design was conducted in Belgium. The MARGA intervention, co-created with parents, incorporated seven interactive sessions over 11 weeks, focussing on parenting practices conceptualised within the Self-Determination Theory and behaviour change methods such as planning and goal setting. Participants included preschoolers (2.5-6 years) and one parent per child (*n* = 141; 49 intervention, 92 control). The primary outcomes were changes in preschoolers’ 24-hour movement behaviour composition and guideline compliance measured using accelerometers and proxy-reported diary. Secondary outcomes included changes in proxy-reported parents’ parenting practices. Both intention-to-treat (ITT) and per-protocol (PP) analyses were performed. In addition, intervention implementation was investigated.

**Results:**

The intervention showed no significant impact on the overall 24-hour movement behaviour composition, nor on 24 h-movement behaviour guideline compliance. However, favourable intervention effects were observed for screen time guideline compliance (ITT: d = 1.5, *p* = 0.04, PP: d = 8.6, *p* = < 0.001) and combinations of screen time and physical activity (ITT: d = 1.2, *p* = 0.05, PP: d = 1.7, *p* = 0.04) or sleep (PP: d = 1.7, *p* = 0.05) guideline compliance. Parenting practices also improved in parents from the intervention condition compared to parents from the control condition, including setting screen time rules (ITT: d = 0.79, *p* = 0.01, PP: d = 1.1, *p* = 0.001), providing choices within sleep routines (ITT: d = 0.63, *p* = 0.04, PP: d = 0.68, *p* = 0.05), parent and preschooler performing physical activity together (PP: d = 0.76, *p* = 0.03) and explaining screen time rules (PP: d = 0.68, *p* = 0.05). Implementation scores indicated moderate engagement, with attendance rates averaging 49.7%.

**Conclusions:**

The intervention showed modest improvements in preschoolers’ 24-hour movement behaviours and parenting practices. Extended follow-up observations might be required to capture changes in preschoolers’ 24-hour movement behaviours, especially considering that the intervention targets preschoolers indirectly by first aiming to influence parents’ parenting practices.

**Trial registration:**

ClinicalTrials.gov (ID NCT06171191).

**Supplementary Information:**

The online version contains supplementary material available at 10.1186/s12966-025-01863-z.

## Background

 A healthy lifestyle is established early in life and tends to persist into adulthood [1]. Sufficient and high quality sleep, low levels of sedentary behaviour and high levels of physical activity form a part of one’s healthy lifestyle [2, 3]. In literature, these behaviours used to be studied mostly as individual behaviours. Within the last decade, a shift in research has occurred towards a more integrated approach [3]. In fact, every activity one conducts during a day can be categorized as either sleep, sedentary behaviour or physical activity. This means that time spent on one behaviour affects the time available for the others within a 24-hour time span. This interrelationship has given rise to the concept of “24-hour movement behaviours” [4] which might yield synergistic effects, where the combined impact on health, e.g. body composition, is greater than the sum of individual changes [3, 5]. Specifically, while an increase in physical activity may be beneficial, the consequential compensatory changes in other behaviours may have beneficial or detrimental effects on health as well [5, 6], e.g., taking time from sedentary behaviour may have different consequences for health than taking time from sleep. This indirect effect of the other behaviours is important to consider when aiming to benefit health [5, 6]. With the rise of this “24-hour movement behaviours” concept, researchers have examined how combinations of movement behaviours relate to health outcomes. For instance, studies show that combinations of higher sleep, lower sedentary behaviour, and higher physical activity have more desirable measures of adiposity and cardio-metabolic health in children and youth (5–17 years) [2]. In both adult and child populations, more recent studies show that for a wide range of health measures, the best outcomes are observed when physical activity is increased at the expense of sedentary behaviour, and to a lesser extent, sleep [6]. For preschoolers, evidence for the combined behavioural impact on health remains limited [7]. Nonetheless, the World Health Organisation (WHO) has recognised the importance of an integrated 24-hour movement behaviour approach and released 24-hour movement behaviour guidelines for early childhood in 2019 [8]. For preschoolers (3–4 years of age), these guidelines recommend 10 to 13 h of good-quality sleep per day, no more than 60 min of sedentary screen time per day and at least 180 min of physical activity per day, including 60 min of higher-intensity activity [8, 9]. The guidelines focus on screen time as a from of sedentary behaviour. Although screen time does not encompass all types of sedentary behaviour, it is associated with overall time spent being sedentary, and excessive screen time has been linked to similar negative health outcomes, such as overweight and obesity [10, 11]. Only a small proportion of preschoolers worldwide (11%) complies with these 24-hour movement behaviour guidelines [9]. Therefore, establishing adequate sleep, minimizing screen time, and increasing physical activity are key public health priorities in this young age group and the potential impact of lifestyle interventions targeting all three behaviours should be explored.

Over the years, numerous interventions have aimed to modify individual sleep, sedentary behaviour, or physical activity in young children, and their effectiveness has been demonstrated in systematic reviews involving children aged 0 to 5 years [12–14]. For instance, sleep interventions increased sleep duration by around nine minutes per night [13], sedentary behaviour interventions reduced sitting time by nineteen minutes per day [12], and physical activity programmes led to a three-minute increase in moderate-to-vigorous physical activity (MVPA) per day [14]. Some interventions have aimed to address sleep, sedentary behaviour, and physical activity simultaneously in preschoolers [15–23]. However, the majority of these studies did not adopt an integrated 24-hour movement behaviour approach that acknowledges the interdependence of behaviours across the day. None of these interventions demonstrated positive effects across all three behaviours [15–22], and most failed to produce significant changes in any of them [15, 18–20, 22]. In contrast, the study of Feng et al. (2024) is, to our knowledge, the only published intervention that targeted the integrated 24-hour movement behaviour compositions in preschoolers [23]. The study was parent-focused internet-delivered intervention lasted 12 weeks and included 147 preschoolers (4.8 ± 0.9 years old, 56.5% boys). Parents received reports about the child’s current level of behaviour compared to the guidelines, educational materials, and online group workshops to teach the parents goal setting, monitoring of behaviours and strategies to overcome barriers in order to improve their preschoolers’ 24-hour movement behaviours. The intervention was successful in reducing preschoolers’ screen time from pretest to post-test and to follow-up (respectively *d* = 0.42 and *d* = 0.27). From pretest to follow-up, both control and intervention group had a decline in physical activity relative to the other behaviours, but the decline was smaller for the intervention group (*d* = 0.59) [23]. These results highlight the potential of an integrated approach to promoting 24-hour movement behaviours in preschoolers. However, further investigation into the effectiveness of integrated interventions focusing on all three 24-hour movement behaviours is warranted.

Preschool-aged children spend a substantial amount of time within the family setting. Within this environment, the critical role of parents in shaping children’s health habits is empirically well-supported and generally accepted within the scientific community [24, 25]. Rhodes et al. (2020) developed an evidence-based consensus statement highlighting the supportive role of the family—including parents—in fostering healthy sleep, sedentary, and physical activity behaviours in children and youth (0–17 years) by encouraging, facilitating, modelling, setting expectations, and engaging in healthy movement behaviours with them. The development of this consensus statement, which involved, for example, expert panels and six reviews, provides a credible and comprehensive overview of current evidence and resources for various stakeholders [25]. It offers a strong rationale to target parents and parental behaviours with an intervention aimed at optimising 24-hour movement behaviours in preschoolers.

In addition, the effectiveness of an intervention may significantly depend on its implementation. An evaluation of the level of implementation can provide valuable insights into the mechanisms and processes driving the observed outcomes and variations in the results [26]. For example, a previous intervention on lifestyle behaviours in children up to 9 years old showed no intervention effect on sedentary time or physical activity [15]. However, groups with lower implementation scores (based on level of implementation and reception) had decreased light physical activity and increased screen time, while those with higher scores maintained stable behaviours [27]. Another intervention study in primary school children showed limited intervention effects on physical activity outcomes. Based on a process evaluation, this was attributed to the lack of encouragement by parents to perform physical activity [28]. Hence, understanding the extent to which the intervention was delivered as intended, the level of participant exposure, and their satisfaction with the intervention is crucial for accurately interpreting the results [29].

The present study aimed to evaluate the effectiveness of the ‘Move ARound And Get Active’ (‘MARGA’) intervention on (1) preschoolers’ 24-hour movement behaviour composition and compliance with the movement behaviour guidelines, and (2) parenting practices, i.e. specific actions parents take to optimise their children’s behaviour [30]. This was investigated for both an intention-to-treat (ITT) group and per-protocol group (PP), compared to a control group. We hypothesized that the intervention would result in improvements in preschoolers’ 24-hour movement behaviour composition, with a sufficient amount of sleep (10–13 h), reduced sedentary behaviour, and increased physical activity. Additionally, we anticipated that significantly more preschoolers would comply with the 24-hour movement behaviour guidelines post intervention, and that parents would experience an improvement in using favourable parenting practices in the intervention condition compared to no changes in the control condition. We furthermore hypothesized that participants with a higher implementation score would experience more favourable effects.

## Methods

### Study design

A two-armed, non-equivalent pretest post-test control group design was conducted in Flanders, Belgium. The MARGA intervention aimed to optimise 24-hour movement behaviours among preschoolers. Ethical approval was provided by the Ethics Committee of Ghent University Hospital (ONZ-2023-0229). The study protocol was registered at ClinicalTrials.gov (ID NCT06171191). The CONSORT checklist and TIDieR checklist were followed and can be found in part A of the supplementary file [31, 32].

### Procedure

This study focused on preschoolers (2.5 to 6 years old) and one of their parents. To be eligible for inclusion, preschoolers had to be without mental or physical limitations (e.g. intellectual disability or paralysis) that may obstruct adherence to the WHO 24-hour movement behaviour guidelines and be able to understand Dutch. The participating parent had to be without mental limitations (e.g. intellectual disability) that could hinder participation in the study and familiar with written and spoken Dutch. The required sample size was estimated with GPower 3.1.9.2 software for two groups (control and intervention condition) and two measurements (pretest and post-test). Based on the reported effect sizes of previously implemented interventions for preschoolers on sleep, sedentary behaviour or physical activity [33, 34], a minimally detectable standardized effect size of f = 0.40, power = 0.80 and alpha = 0.05, was intended. The a *priori* power analysis suggested a total sample of 53 parent-child dyads, accounting for a 20% dropout rate.

Convenience sampling was applied to recruit participants from October 2023 to February 2024. In total, 143 preschools within a practical distance from Ghent University Hospital (± 40 km) were contacted by phone call and email to ask whether the preschool wanted to participate in the study as a source to recruit participants. Initially, preschools were targeted with at least 50% of students having low levels of parental education, a proxy for Socio-Economic position (SEP) [35, 36]. This approach was used due to the challenge of reaching preschoolers with low SEP, prompting additional efforts to ensure their inclusion. Due to insufficient response rates, this criterion was gradually decreased to 30%, then 25%, and eventually the call was opened to all preschools within the specified radius to ensure a sufficiently large sample. Consequently, the final sample may reflect a higher proportion of children from advantaged socio-economic contexts than initially intended. To prevent stigmatization, all preschoolers within the selected preschools were given the opportunity to participate, including those with a higher SEP. Before the call, each preschool was assigned to either an intervention or control condition to inform the preschool clearly about the expectations when participating in the study and for practical considerations, such as ensuring participants in preschools within the same area were assigned to the same group to facilitate feasibility of intervention delivery and avoid contamination between schools. Schools offer the advantage of being settings where large numbers of children can be reached simultaneously. However, a key limitation is that children within a school cannot be randomly assigned to intervention or control groups, as they may influence one another. This constraint led to the adoption of a quasi-experimental design rather than a randomised controlled trial. Due to the challenging recruitment process, no matching was performed between intervention and control schools. However, efforts were made to ensure a similar distribution across both conditions in terms of urban and rural settings, as well as school size (i.e., schools with larger and smaller pupil populations).” In participating preschools, preschoolers’ parents were recruited via two methods, depending on the preschool’s method of operation: either via a flyer in the preschoolers’ book bags, or via an online flyer with a link to a Google Form for enrolment. The enrolment process required participants (i.e., parents and preschoolers) to provide their name, email, and phone number. Afterwards, interested parents were contacted via phone call or email to receive additional details about the study and asked about their willingness to participate. If parents were willing to participate, they were offered an Informed Consent Form, which was expected to be signed before the start of the first measurement. The researcher ensured a duplicate copy of the signed form was retained on paper. Measurements took place at the preschool and parents were notified of the measurement dates by email.

### The ‘MARGA’ intervention

**Fig. 1:**
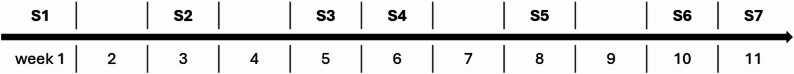
Timeline of the total intervention period

The MARGA intervention was developed through co-creation with parents. The Intervention Mapping Protocol (IMP) was used throughout the co-creational process to provide theory- and evidence-based guidance for the development of the intervention [37, 38]. The Self-Determination Theory was imbedded in the intervention as a foundational model to intrinsically motivate a preschooler towards healthy behaviour by supporting three basic psychological needs through parenting practices [39]: (1) autonomy by allowing a child to make choices, (2) relatedness by providing warmth, i.e., showing interest in the child’s behaviour or performing the behaviour together, and (3) competence by providing adequate structure such as clear rules [24]. A detailed description of the MARGA intervention development is provided elsewhere (“preprint citations”). The MARGA intervention consisted of seven sessions (of 1 h and 30 min) that were implemented between February 2024 until May 2024. A timeline of the total intervention period can be found in Fig. 1. Each intervention group (4 groups in total) consisted of maximum 12 parents and their preschool child. The sessions took place at participating preschools which had the facility to organise some activities using sports equipment and where a slideshow could be presented. An overview of the intervention themes for each session and the behaviour change methods per session can be found in Table 1. Intervention materials used throughout the sessions included an introductory Power Point presentation on 24-hour movement behaviours and SDT, a physical activity calendar with inspirational activities to plan, games to limit screen time, a material box to exchange toys each session as an alternative to screen time, a week schedule, tips and tricks collected from parent group members during the support group sessions, a sleep routine schedule, a list of physical activity games that were tried out during the sessions, tools to sustain behavioural change or deal with relapse (e.g., an if-then plan template for problem solving). The content of session 1, 3, 6 and 7 was only directed to parents. These included informational sessions and support groups in which parents could share experiences. The content was specifically tailored to the experiences and concerns of parents and not age-appropriate or engaging for preschoolers. For these sessions, preschoolers could separately try out different sports. All sessions with parents (with or without preschoolers) were facilitated by researcher MD ensuring high fidelity. The sport sessions for preschoolers only were facilitated by Ghent University master’s students of Physical Education and Movement Sciences, Rehabilitation Sciences and Physiotherapy, and Health Promotion. The control group received the intervention materials after all data-collection was completed (after follow-up test (T2)).

Total intervention period of 11 weeks with 7 sessions (S).

**Table 1:** Overview of themes of each intervention session

Session number	Themes	
	Parent	Child
1	**Information session**: - Introduction into 24-hour movement behaviours - Background information about the intervention development - Introduction to the Self-Determination Theory - Alternatives for screen time Behaviour change methods: verbal persuasion, consciousness raising, opportunities for social comparison, mobilizing social support, modelling, facilitation	**Sport**: Introduction to ball sports
2	**Planning session**: - Introduction to week scheduling through interactive story - Parent and child make a week schedule together Behaviour change methods: modelling, direct, experience, goal setting, public commitment, opportunities of social comparison	
3	**Support group**: - Sharing experiences with regard to week scheduling - Practice Self-Determination Theory Behaviour change methods: repeated exposure, guided practices, opportunities for social comparison, mobilizing social support, planning coping responses/problem-solving	**Sport**: Introduction to dance
4	Activity games with common home equipment Behaviour change methods: modelling, direct experience, facilitation	
5	**Planning session**: - Introduction to sleep routine through interactive story - Parent and child make a sleep routine together Behaviour change methods: modelling, direct, experience, goal setting, public commitment, opportunities of social comparison	
6	**Support group**: - Sharing experiences with regard to sleep routine Behaviour change methods: repeated exposure, guided practices, opportunities for social comparison, mobilizing social support, planning coping responses/problem-solving	**Sport**: Introduction to gymnastics
7	**Information session**: - Workshop on how to maintain healthy behaviour and how to deal with relapse Behaviour change methods: repeated exposure, guided practices, modelling, self-monitoring, planning coping responses, graded tasks	**Sport**: Introduction to athletics

### Measures

Measurements took place at two timepoints: the pretest (T0) from December 2023 until the beginning of March 2024 and the post-test (T1) from May 2024 until the end of June 2024.

#### Demographic variables

At T0, preschoolers’ and parents’ age and sex and parents’ highest educational degree were self-reported in an online questionnaire in REDCap 14.0.35 [40, 41], an electronic data capture tool hosted at Ghent University Hospital. The reported educational level of the parent who completed the questionnaire was used as a proxy for SEP [35, 36]. Lower SEP was determined as parents having no higher education and higher SEP as parents having higher education of at least a professional bachelor or equivalent [42].

At T0 and T1, body weight and height were measured during a preschool visit according to a standardized protocol based on previous research within the research team [43]. Preschoolers were measured with bare feet and wearing light clothing. Body height was measured to the nearest 0.1 cm by means of the SECA 225 or SECA 214 Leicester Portable stadiometer (Seca, Hamburg, Germany). Body weight was measured to the nearest 0.1 kg by a calibrated electronic scale SECA 861 or SECA 813. For these parameters, two measurements were conducted and the mean was used for analyses. If the readings differed by more than 1%, a third measurement was obtained and the mean of the two measurements with the smallest difference was used. Preschoolers’ Body Mass Index (BMI; kg/m^2^) z-scores were calculated on the basis of Anthro version 3.2.2 and AnthroPlus version 1.0.4 software (WHO, Geneva, Switzerland). Sex- and age-specific BMI z-scores provide a relative measure of adiposity adjusted for age and sex. The z-score is the number of standard units that a person’s BMI deviates from a mean or reference value. Parents’ body weight and height were self-reported through the online questionnaire and used to calculate parents’ BMI.

#### Primary outcome: 24-hour movement behaviours

At T0 and T1, preschoolers’ sleep duration, sedentary behaviour, light physical activity (LPA) and moderate-to-vigorous physical activity (MVPA) were measured using ActiGraph accelerometers (GT3X+, GT3X BT, ActiGraph, Pensacola, FL). Preschoolers were fitted with the accelerometer after the anthropometric measurements at the preschool. The accelerometers were fitted on the right hip at the right mid-axillary line using an adjustable elastic belt. Preschoolers received written instructions, including pictures, for their parents on how to use the accelerometer and when the devices would be collected at preschool. Participants were instructed to wear the accelerometer continuously for seven days and nights, and to only remove it for water-based activities. Accelerometer data were assessed at a sampling frequency of 30 Hz and activity counts were aggregated into 15-second epochs. All accelerometer data were processed in R via GGIR version 3.1.1 [44]. Non-wear time was defined as 20 min of consecutive zero accelerometer output counts. To be included in the study, preschoolers had to provide at least three days including one weekend day of valid accelerometry data with a minimum wear time of 16 h/day [45, 46]. Belgian public holidays that occurred during measurement periods were considered as weekend days. Time spent in sleep, sedentary behaviour, LPA and MVPA was averaged for all valid days (minutes/day) weighted for weekend- and weekdays (((weekend day x 2) + (weekday x 5))/7) and used in the analysis. For sleep, the Sadeh1994 algorithm was used [45, 47]. To differentiate sedentary behaviour from LPA, and LPA from MVPA, cut-points of 25 and 420 activity counts/15 seconds were used, respectively [48–50].

In addition to wearing the accelerometer, the parent completed a diary for each of the seven days. This diary collected data about the time their preschool child fell asleep, woke up, start and end times of naps, start and end times of non-wear time, minutes of screen time (e.g., television viewing, use of tablets, smartphone, computer, game consoles) and whether the preschooler was at preschool, home and/or sick. Sick days were excluded. Falling asleep, waking up, nap times and non-wear times were used in a sleep log to support GGIR processing of the accelerometer data. Nonetheless, diaries can be subject to recall bias and social desirability bias [51]. To mitigate this, clear instructions were provided, and in cases where no valid diary data were available, default GGIR settings were applied [44, 52].

The 24-hour movement behaviours were operationalised and used in terms of 24-hour movement behaviour compositions (sleep, sedentary time, LPA and MVPA) and compliance with the 24-hour movement behaviour guidelines. The 24-hour movement behaviour compositions were analysed using Compositional Data Analysis (CoDA). CoDA allows all 24-hour movement behaviours to be analysed simultaneously, accounting for their co-dependence within a finite time frame [53]. This approach facilitates an understanding of how changes in time spent on the behaviours affect the overall 24-hour movement behaviour composition. By using CoDA, interventions can be evaluated to determine whether they lead to more optimal movement behaviour compositions, characterized by a sufficient amount of sleep, reduced sedentary time, and increased physical activity [3, 5]. To apply CoDA, time-use behaviours were expressed as a set of isometric log-ratios (ILR). Prior to the log-transformations the presence of zero values in the 24-hour movement behaviours was carefully examined, which is necessary to enable the transformations. No zero values were observed.

Compliance with the 24-hour movement behaviour guidelines was derived from accelerometer data for physical activity and sleep duration, in combination with data from the diary for screen time, averaged for all valid days (minutes/day) weighted for weekend- and weekdays (((weekend day x 2) + (weekday x 5))/7). Preschoolers were classified as complying with the 24-hour movement behaviour guidelines when: (a) physical activity: total physical activity ≥ 180 min/day and MVPA ≥ 60 min/day; (b) screen time: ≤ 60 min/day and (c) sleep duration: 10–13 h/day [8]. Compliance with each individual guideline and all other possible combinations were also calculated. Although the age range in this study (2.5–6 years) was broader than the age range for which the WHO 24-hour movement guidelines for preschoolers (3–4 years) were developed, we chose to focus solely on these guidelines: (1) to provide participants with a clear and consistent message, and (2) to facilitate interpretation in the analyses. This approach has also been adopted in previous research examining guideline compliance among preschoolers [54–57].

#### Secondary outcome: parenting practices

At T0 and T1, parenting practices were derived from the online questionnaire. Table 2 provides an overview of the questions used for further analyses structured following the basic needs of the Self-Determination Theory [39]. Answer options were “1; never”, “2; seldom”, “3; sometimes”, “4; often”, “5; always” or “6; not applicable”. For further analyses in this study “not applicable” was recoded into “never”. The questions were adapted from the Movie Models questionnaire, which was a questionnaire developed for the Movie Models intervention, which also targeted specific parenting practices [58]. This questionnaire was based on the validated Parental Support for Physical Activity Scale (Cronbach’s alpha = 0.78; test–retest reliability: *R* = 0.81) [59], as well as the Parenting Strategies for Eating and Activity Scale (Cronbach’s alpha = 0.81–0.82; test–retest reliability: *R* = 0.81) [60].For parenting practices, there was also a follow-up measurement (T2) in October and November 2024. For ease of interpretation, these are not presented in the main text. However, results including all three measurements (T0, T1 and T2) of parenting practices can be found in Table D1 in supplementary file.

**Table 2:** Questions to assess parenting practices that support the needs of the Self-Determination theory

Parenting practices based on SDT needs	Questions per behaviour		
	PA	ST/SB	Sleep
Autonomy support	I let my child choose for themselves which form of movement activity they want to do (PA choice)	I provide my child with alternatives to screen activities (e.g., colouring, board games, etc.). (ST alternatives)	I let my child make choices during his/her sleep routine (e.g., which pyjamas, which story, which stuffed animal, etc.). (choice in sleep routine)
Positive involvement, warmth	I give my child a compliment when he/she does a movement activity (PA compliment)	I explain to my child why there are rules about screen activities. (ST rules explanation)	
	I move together with my child. (PA together)		
Competence support, structure	I regularly schedule a movement activity for my child (e.g., a biweekly activity). (PA plan)	In our family, there are rules for my child regarding screen activities (TV, Netflix, Disney+, video games, YouTube, etc.) (ST rules)	My child goes to bed at a fixed time (fixed bedtime)
		I monitor how much time my child spends on screen activities. (monitor ST)	My child has fixed habits for bedtime, a sleep routine (e.g., reading a story, brushing teeth, a religious activity, drinking milk, giving a kiss, etc.). (sleep routine)

*SDT* Self-Determination Theory, *ST* screen time, *SB* sedentary behaviour, *PA* physical activity

#### Intervention implementation

The participants of the intervention condition were assigned an implementation score. Participants above the median of this score were considered the PP group. For clarity, the ITT group were all participants who had at least valid demographic data and valid data for primary or secondary outcomes at one of the timepoints, and included the PP group. The implementation score was derived from three components of the process evaluation framework of Saunders et al. (2005): (1) dose delivered (i.e. was the intervention delivered by the researchers?), (2) dose received – exposure (i.e. was the intervention received by the participants?), and (3) dose received – satisfaction (i.e. how was the intervention received by the participants?) [26]. This score was calculated using responses from logbooks (L) of the researchers, process evaluation questionnaires completed by participants at the end of each session (session feedback, SF) and at T1 (post process evaluation, PPE). The specific questions, response options and coding can be found in Table H1 in supplementary. For ease of interpretation, all scores were rescaled to a 10-point scale. A higher score indicates better implementation.

### Data analyses

All analyses were conducted in R version 4.4.1 [61] and a significance level of alpha ≤ 0.05 was used.

Binary logistic regression analyses were conducted to examine potential systematic differences in group, child and parent age, child and parent sex, child BMI-z score, parent BMI, and parent educational level between participants with complete valid data at both T0 and T1 and those with valid data at T0 but invalid or missing data at T1. A similar analysis was conducted for T0 to T2 (see Table B1 in supplementary file).

Descriptive sample characteristics at T0 were provided. Continuous variables were summarised by means and standard deviations and categorical variables by counts and percentages. Independent two-sample t-tests for continuous variables and chi-square tests for categorical variables were used to compare participants from the intervention and control condition. Descriptive statistics of the composition were presented as central tendency. We calculated geometric means of each behaviour, which ensures the proportional relationship among the behaviours is reflected. After calculating these means, a linear adjustment was made so that the behaviours collectively sum to 1440 min (total number of minutes within 24 h), and compositional log-ratio variation was described using a matrix of all possible pairwise log-ratios variations [62]. To analyse compositional data, the compositions package was used [63, 64].

Mixed models were performed to evaluate the intervention effects using the lme4 package [65]. Of primary interest was the difference between the intervention and control condition from T0-T1 (i.e., difference of differences) on 24-hour movement behaviour compositions, guideline compliance and parenting practices, retrieving the interaction effect as the main result. For the compositions and parenting practices these were linear mixed models and for guideline compliance these were mixed model logistic regressions. Time by Group (intervention versus control) interaction terms were reported for T1-T0 changes in outcome variables. The intervention-effects were presented as effect size (Cohen’s d), which was categorised as small, medium, and large, corresponding to 0.2, 0.5, and 0.8, respectively [66–69]. For the logistic regressions a formula for approximating Cohen’s *d* was used [70, 71]: *d* = ln(*OR*)/1.81.

The models were performed as ITT analysis (compared to control condition), in which all participants of the intervention group were included, and as PP analysis (compared to control condition), based on the implementation score. In the supplementary file (D.), results can also be found for a third follow-up time point (T2) for parenting practices. For ease of interpretation, these results are not reported in the main paper.

The Likelihood Ratio Test (lme4 package [65]) was performed to check the need for clustering for preschool. Because some of the models on parenting practices had a better fit (*p* < 0.05) when clustering was accounted for by including random intercepts at the preschool level, all models were clustered for preschool to be consistent. Post hoc intraclass correlation coefficients (ICCs) were estimated to provide insight into the variance explained by the random intercepts at the preschool level (see Table D1 in the supplementary file). No covariates were included to the final models. However, all descriptive characteristics reported were considered as potential covariates. They were all added to the models to assess whether they had an impact on the potential intervention effect. Akaike information criterion (AIC) was used to check the best model fit comparing with to without the covariates [72]. The final models fit was assessed via diagnostic plots of linearity, normality of residuals and homogeneity of variance [68]. All model estimates and p-values were reported without adjustment for multiple comparisons, allowing to interpret the results while acknowledging the trade-off between Type II error and Type I error [73–75].

## Results

### Participant flow and drop-out analyses

**Fig. 2:**
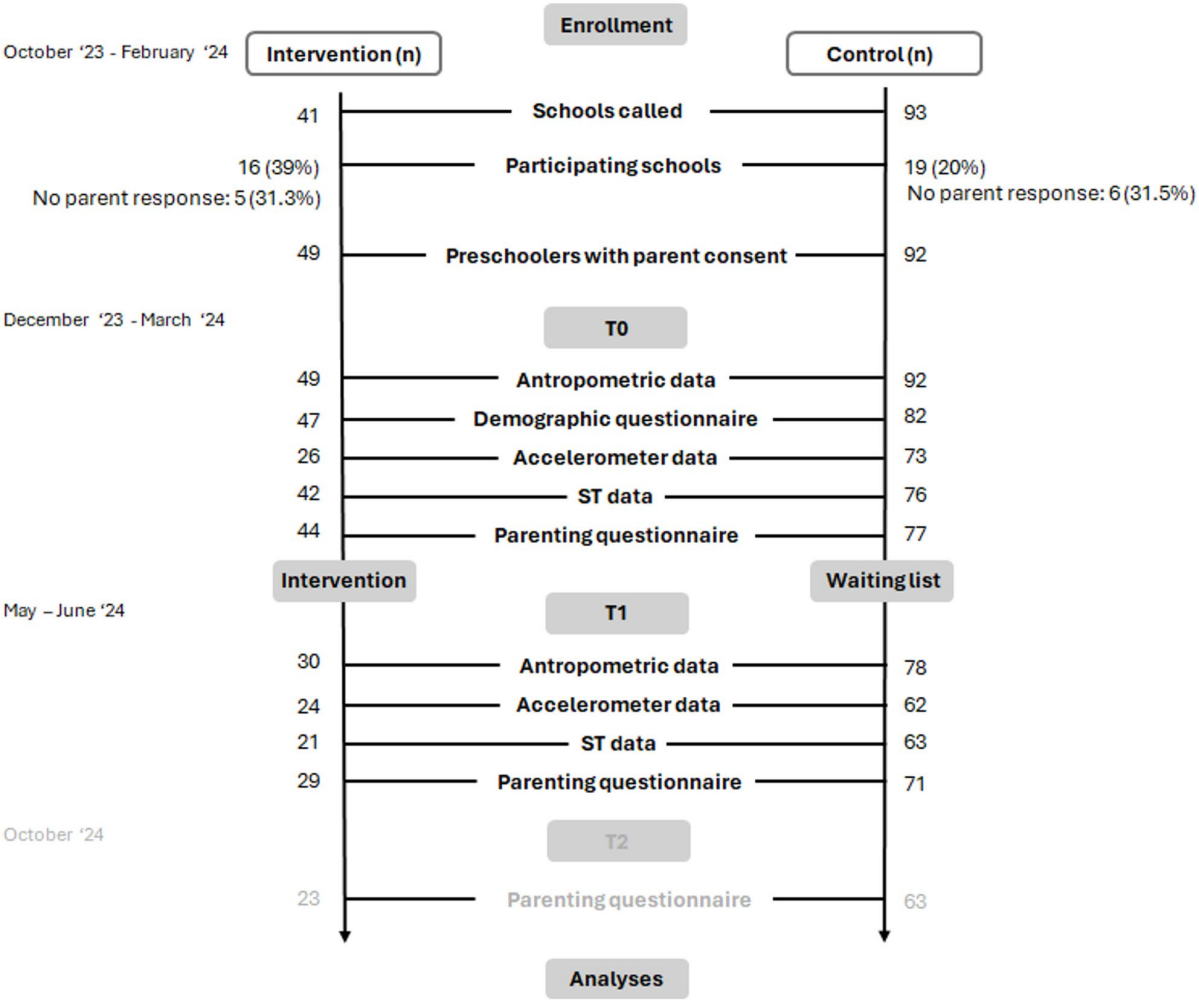
Participant flow. n = number, ST = screen time, T0 = pretest, T1 = post-test, T2 = follow-up test.

Figure 2 shows the participant flow. In total, 41 intervention preschools and 93 control preschools were invited to participate. Of these, 39% intervention preschools (*n* = 16) and 20% control preschools (*n* = 19) agreed to participate. In Fig. 2, these are named “participating schools”. A total of 141 parents signed the informed consent form and agreed to participate of which 49 for the intervention group and 92 in the control group.

The odds of having valid data at T0, but invalid or missing data at T1 are presented in Table B1 in the supplementary file. For the primary outcomes—24-hour compositions and guideline compliance—no significant differences were found between participants with complete data at T0 and T1 and those with missing or invalid data at T1. This suggests that data availability at T1 was not systematically related to participant characteristics. For the secondary outcome, parenting practices, participants in the intervention condition were more likely to have missing or invalid data at T1 compared to those in the control condition (OR [CI]: 4.0 [1.6; 11.0]). For the sociodemographic variables, participants with a higher parental educational level were less likely to have missing or invalid data at T1 than those with a lower level (OR [CI]: 0.3 [0.1; 0.8]).

### Descriptive characteristics

Table 3 represents the descriptive characteristics of the participants at T0 for the sample used for the primary outcomes. The descriptive characteristics for the secondary outcomes can be found in supplementary (Table C1). There were no significant differences for sample characteristics between the control condition and intervention condition. Respectively for the control condition, the ITT group and the PP group, the average age of the preschoolers was 4.2 (SD = 0.9), 4.0 (SD = 0.9) and 4.2 (SD = 1.0). In total 43.8%, 38.5% and 52.6% were female preschoolers. For the parents, 75.3%, 84.6% and 84.2% were mothers and 89%, 84.6% and 84.2% had a higher educational level.

**Table 3:** Descriptive characteristics of participants – sample for the primary outcomes

	control	ITT	PP	*p*-value (C vs. ITT)	*p*-value (C vs. PP)
*N*	73	26	19		
Age child in years (mean (SD))	4.2 (0.9)	4.01 (0.9)	4.2 (1.0)	0.47	0.73
Age parent in years (mean (SD))	36.2 (4.5)	36.2 (3.9)	36.9 (3.9)	0.95	0.56
Sex child = female (%)	32 (43.8)	10 (38.5)	10 (52.6)	0.81	0.67
Sex parent = female (%)	55 (75.3)	22 (84.6)	16 (84.2)	0.48	0.61
BMI z-score child (mean (SD))	0.4 (0.8)	0.66 (1.0)	0.5 (1.1)	0.13	0.41
BMI parent (mean (SD))	23.3 (3.2)	23.81 (4.5)	23.6 (4.8)	0.53	0.78
Parental education level = higher (%)	65 (89.0)	22 (84.6)	16 (84.2)	0.81	0.86

*N* number, *SD* standard deviation, *BMI* Body Mass Index, *ITT* intention-to-treat, *PP* per-protocol, *C* control

### Intervention-effects 

#### 24-hour movement behaviour composition

For the 24-hour composition, there was no difference in the change in 24-hour movement behaviour composition from T0 to T1 between the control condition and the intervention condition for both ITT (numerator Degrees of Freedom (numDF) = 3, denominator Degrees of Freedom (denDF) = 519, F = 0.5, *p* = 0.70) and PP (numDF = 3, denDF = 735, F = 0.9, *p* = 0.47). Table 4 shows the descriptive statistics of the compositional data. Mean differences were not provided as a composition should be interpreted as a whole. In addition ilr mean differences did not have an added value for interpretation of the results.

**Table 4:** Descriptive statistics for 24-hour movement behaviour compositional data

Outcomes	Group	Descriptive statistics	
		T0	T1
N	ITT	26	24
	PP	19	19
	C	73	62
		Mean* (minutes)	
Sleep	ITT	629	623
	PP	622	616
	C	622	606
SB	ITT	395	375
	PP	401	384
	C	416	401
LPA	ITT	333	350
	PP	335	348
	C	319	333
MVPA	ITT	83	92
	PP	83	92
	C	82	100

*ITT* Intervention intention-to-treat group, *PP* intervention per-protocol group, *C* Control group, *SB* sedentary behaviour, *LPA* light intensity physical activity, *MVPA* moderate to vigorous intensity physical activity, *T0* pretest, *T1* post-test

*SD is not applicable for Compositional Data Analysis (CoDA), dispersion is represented by variation matrices Tables E1-E6 in supplementary file

#### 24-hour movement behaviour guideline compliance

Table 5 shows the intervention effects for compliance with the 24-hour movement behaviour guidelines. There was no difference in the change in proportion of preschoolers complying with all the guidelines from T0 to T1 between the conditions (ITT: *p* = 0.24, PP: *p* = 0.11). However, there were between-group differences for changes in compliance to individual guidelines. The intervention condition had higher odds than the control condition for becoming compliant with the screen time guideline (ITT: *d* = 1.5, *p* = 0.04, PP: *d* = 8.6, p = < 0.001), and with the combination of the sleep and screen time guidelines (ITT: *d* = 1.2, *p* = 0.05, PP: *d* = 1.7, *p* = 0.04). The intervention condition also had higher odds of becoming compliant with the combination of screen time and physical activity guidelines than the control condition, but only in PP analyses (*d* = 1.7, *p* = 0.05). The intervention condition had lower odds of becoming compliant with the physical activity guideline (ITT: *d*=-6.8, *p* = 0.01, PP: *d*=-5.9, *p* = 0.04) compared to the control condition.

**Table 5:** Means, percentages, interaction effects and changes in outcomes from T0 to T1 for 24-hour movement behaviour guideline compliance

Outcomes	Group	Descriptive statistics		Mixed model results T1-T0			
		T0	T1	Time x Group Ref. = control group and pretest			
N	ITT	24	18				
	PP	19	15				
	C	66	56				
		% guideline compliance		Groups	OR [95%CI]	p-value	Effect size
All	ITT	16.7	44.4	C-ITT	3.5 [0.4, 26.7]	0.24	0.7
	PP	15.8	46.7	C-PP	11.9 [0.6, 253.6]	0.11	1.4
	C	31.8	39.3				
Sleep + ST	ITT	20.8	55.6	C-ITT	8.9 [1.0, 78.2]	0.05	1.2
	PP	21.1	53.3	C-PP	20.6 [1.1, 394.4]	0.04	1.7
	C	39.4	39.3				
Sleep + PA	ITT	62.5	50.0	C-ITT	0.5 [0.1, 2.4]	0.39	-0.4
	PP	57.9	46.7	C-PP	0.5 [0.1, 2.8]	0.43	-0.4
	C	51.5	55.4				
ST + PA	ITT	16.7*	61.1	C-ITT	5.0 [0.6, 38.6]	0.12	0.9
	PP	15.8*	66.7	C-PP	23.5 [1.0, 546.8]	0.05	1.7
	C	51.5	67.9				
Sleep	ITT	66.7	61.1	C-ITT	1.0 [0.2, 5.0]	0.99	0.1
	PP	63.2	53.3	C-PP	0.8 [0.1, 4.4]	0.79	-0.1
	C	59.1	55.4				
ST	ITT	29.2*	77.8	C-ITT	14.7 [1.2, 179.6]	0.04**	1.5
	PP	31.6*	80.0	C-PP	5.7e + 06 [7.8e + 03, 4.2e + 09]	< 0.001**	8.6
	C	62.1	69.6				
PA	ITT	83.3	83.3	C-ITT	4.6e-06 [3.5e-10, 6.0e-02]	0.01	-6.8
	PP	78.9	86.7	C-PP	2.3e-05 [8.1e-10, 6.4e-01]	0.04	-5.9
	C	87.9	96.4				

One intervention and one control group participant had zero compliance on the pretest, one participant of the control group had zero compliance for the post-test

*ITT* Intervention intention-to-treat group, *PP* intervention per-protocol group, *C* Control group, *ST* screen time, *PA* physical activity, *SE* standard error, *T0* pretest, *T1* post-test, *OR* Odds Ratio, *CI* Confidence Interval, *Ref. *reference category

*Guideline compliance significantly (alpha ≤ 0.05) differed for C group and the ITT or PP group. **Indication for regression to the mean effect (see Supplementary file G.)

Table 6 shows the results for the secondary outcome of parenting practices. The intervention condition had a higher T0 to T1 increase in their score than the control condition in the following practices: allowing their preschooler to choose sleep routine activities (autonomy support) (ITT: *d* = 0.63, *p* = 0.04, PP: *d* = 0.68, *p* = 0.05) and screen time rules (structure) (ITT: *d* = 0.79, *p* = 0.01, PP: *d* = 1.1, *p* = 0.001). The intervention condition also had a higher increase for performing physical activity together and for explaining screen time rules (warmth), but only in PP analyses (respectively, *d* = 0.76, *p* = 0.03; *d* = 0.68, *p* = 0.05). 

**Table 6:** Means, percentages, interaction effects and changes in outcomes from T0 to T1 for parenting practices

Outcomes	Group	Descriptive statistics		Mixed model results T1-T0			
		T0	T1	Adjusted mean difference (intervention vs. control) Estimate (SE) [95%CI]		Time x Group p-value	Effect size
N	ITT	44	29				
	PP	22	21				
	C	77	71				
		Adjusted mean (SD)**					
1: Autonomy support							
PA choice	ITT	3.9 (0.1)	4.0 (0.1)	ITT-C	-0.01 (0.2) [-0.3; 0.3]	0.97	(-0.10)
	PP	4.0 (0.2)	4.0 (0.2)	PP-C	-0.1 (0.2) [-0.4; 0.3]	0.66	(-0.14)
	C	4.1 (0.1)	4.1 (0.1)				
ST alternatives	ITT	4.1 (0.1)	4.3 (0.1)	ITT-C	0.2 (0.2) [-0.2; 0.6]	0.32	(0.29)
	PP	4.2 (0.2)	4.5 (0.2)	PP-C	0.2 (0.2) [-0.2; 0.7]	0.29	(0.37)
	C	4.3 (0.1)	4.3 (0.1)				
choice in sleep routine	ITT	4.1 (0.1)	4.5 (0.2)	ITT-C	0.4 (0.2) [0.03; 0.8]	0.04	(0.63)
	PP	4.2 (0.2)	4.6 (0.2)	PP-C	0.5 (0.2) [-0.0; 0.9]	0.05	(0.68)
	C	4.3 (0.1)	4.2 (0.1)				
2: Warmth							
PA compliment	ITT	3.9 (0.2)	4.2 (0.2)	ITT-C	0.1 (0.2) [ -0.3; 0.5]	0.50	(0.20)
	PP	4.2 (0.2)	4.4 (0.2)	PP-C	-0.1 (0.2) [-0.5; 0.4]	0.77	(-0.10)
	C	3.8 (0.1)	4.0 (0.1)				
PA together	ITT	3.3 (0.1)	3.7 (0.1)	ITT-C	0.3(0.2) [-0.04; 0.6]	0.09	(0.52)
	PP	3.3 (0.2)	3.8 (0.2)	PP-C	0.4 (0.2) [0.04; 0.8]	0.03	(0.76)
	C	3.5 (0.1)	3.6 (0.1)				
ST rules explanation	ITT	3.4 (0.2)	3.7 (0.2)	ITT-C	0.4 (0.2) [-0.1; 0.8]	0.11	(0.49)
	PP	3.4 (0.3)	3.9 (0.3)	PP-C	0.5 (0.3) [-0.01; 1.0]	0.05	(0.68)
	C	3.4 (0.2)	3.4 (0.2)				
3: Structure							
PA plan	ITT	3.3 (0.2)	3.2 (0.2)*	ITT-C	-0.2 (0.3) [-0.7; 0.3]	0.51	(-0.20)
	PP	3.2 (0.3)	3.2 (0.3)*	PP-C	-0.1 (0.3) [-0.7; 0.5]	0.65	(-0.16)
	C	3.6 (0.2)	3.7 (0.2)				
ST rules	ITT	3.4 (0.2)*	4.0 (0.2)	ITT-C	0.6 (0.2) [0.2; 1.1]	0.01	(0.79)
	PP	3.1 (0.3)*	4.0 (0.3)	PP-C	0.9 (0.3) [0.4; 1.4]	0.001	(1.1)
	C	3.8 (0.2)	3.8 (0.2)				
monitor ST	ITT	2.8 (0.2)	3.3 (0.2)	ITT-C	0.5 (0.3) [-0.03; 0.9]	0.07	(0.56)
	PP	2.9 (0.3)	3.4 (0.3)	PP-C	0.5 (0.3) [-0.06; 1.0]	0.08	(0.59)
	C	3.3 (0.2)	3.3 (0.2)				
fixed bedtime	ITT	4.3 (0.1)	4.1 (0.1)*	ITT-C	-0.2 (0.2) [-0.5; 0.1]	0.30	(-0.31)
	PP	4.1 (0.2)	4.0 (0.2)*	PP-C	-0.1 (0.2) [-0.5; 0.3]	0.55	(-0.21)
	C	4.3 (0.1)	4.3 (0.1)				
sleep routine	ITT	4.7 (0.1)	4.6 (0.1)	ITT-C	-0.1 (0.1) [-0.2; 0.1]	0.57	(-0.17)
	PP	4.7 (0.1)	4.5 (0.1)	PP-C	-0.1 (0.1) [-2.9e-01; 0.1]	0.36	(-0.33)
	C	4.8 (0.1)	4.7 (0.1)				

*ITT* Intervention intention-to-treat group, *PP* intervention per-protocol group, *C* Control group, *ST* screen time, *PA* physical activity, *SE* standard error, *SD* standard deviation, *T0* pretest, *T1* post-test, *CI* Confidence Interval

*Parenting practice score significantly (alpha ≤ 0.05) different for C group vs. ITT or PP group.**The means and mean differences are model-adjusted

### Intervention implementation 

On average, participants attended 49.7% (*n* = 22) of the group sessions, with a minimum session attendance of 43.2% and a maximum attendance of 65.9%. Dose delivered had an average score of 5.4/10 (SD = 3.8), dose received-exposure had an average score of 3.8/10 (SD = 3.6), and dose received-satisfaction had an average score of 5.0/10 (SD = 3.9). The overall average implementation score was 4.7/10 (SD = 3.7). The most used intervention material was the week schedule with 59.1% (*n* = 26) of participants who used it, and 45.5% (*n* = 20) of participants who used it frequently. The content of session 5, focussing on sleep routine, was perceived the least new, with 11.4% (*n* = 5) of participants scoring it as new. More details with regard to the implementation score can be found in table supplementary Table F1.

## Discussion

The present study examined the effectiveness of an intervention to optimise preschoolers’ 24-hour movement behaviours (primary outcome) and parenting practices (secondary outcome), analysed for both ITT and PP groups. We hypothesized that, compared to the control condition, the intervention condition would show improvements in 24-hour movement behaviour composition, increased compliance with 24-hour movement behaviour guidelines, and positive changes in parenting practices, with stronger effects in the PP analyses.

Contrary to our hypothesis, no intervention effect was found for the 24-hour movement behaviour composition, nor for complying with the integrated 24-hour movement behaviour guidelines. The previous similar parent-focused intervention of Feng et al. (2024) also did not find intervention effects for the integrated 24-hour movement behaviour composition [23]. Although developed independently, our intervention shared several similarities with the intervention study conducted by Feng et al. (2024). Apart from both being parent-focused and aiming to improve integrated 24-hour movement behaviours in preschoolers, both interventions shared behaviour change strategies such as goal setting and planning, and applications such as planning schedules and inspiration for increasing physical activity and reducing screen time. Group workshops were also a common feature, although Feng et al. (2024) conducted theirs online due to the COVID-19 pandemic. Also, the intervention of Feng et al. (2024) was limited to three group workshops and mainly existed of sending educational materials via e-mail [23, 76]. In contrast, parents involved in the co-creation of our intervention expressed a preference for in-person meetings. They valued shared experiences, such as trying-out movement games together and learning from other parents and preschoolers. They emphasized the importance of real-life connectedness, seeking trust-building and informal social support. Consequently, the behaviour change method “mobilizing social support” was not included in Feng et al. (2024), whereas our study explicitly addressed this parental need. Notably, Feng et al. (2024) did not involve parents in the development process [23, 76]. However, it is unclear how these similarities and differences contributed to the lack of an integrated 24-hour movement behaviour intervention effect. Potentially the time-frame of both interventions might have been too short to observe effects on all three behaviours: our intervention lasted 11 weeks, and the intervention by Feng et al. (2024) lasted 12 weeks. As such, Feng et al. (2024) found that movement behaviours of preschoolers -both in the control and intervention condition- changed unfavourably over a 24-week period, which corresponded to the time of their follow-up measurement. Both conditions had a decline in physical activity relative to the other behaviours. However, the decline was less pronounced in the intervention condition, which partially supports their hypothesis that integrated approach is effective in improving preschoolers’ overall movement behaviours [23]. In our study, longer follow-up data on 24-hour movement behaviours were not available for comparison.

Although there were no significant effects for the integrated 24-hour movement behaviours, associations for guideline compliance with individual behaviours still provide relevant information and prevent valuable insights from being missed. Similar to our findings, the intervention of Feng et al. (2024) was effective in reducing preschoolers’ screen time from T0 to T1. We found a favourable intervention effect on compliance with the screen time guideline, as well as the combination of the screen time guideline and the sleep guideline in both ITT and PP analyses, and the combination of the screen guideline and the physical activity guideline in PP analyses. These findings were probably driven mainly by the screen time guideline. This is because no favourable intervention effect was observed for individual compliance with the sleep or PA guidelines. However, cautious interpretation of the PP analyses for screen time guideline compliance is warranted, as the wide confidence interval raises questions about the robustness of the model. We also observed some intervention effects for screen time-related parenting practices, which might potentially underly the effect on screen time guideline compliance. There was a medium effect size for parents setting screen time rules for their preschooler for both ITT and PP analyses and a small effect size for explaining the rationale behind screen time rules for PP analyses. All of these screen time related effects seemed to have larger effect sizes for the PP group than for the ITT group, suggesting that the intervention program itself contributed to the effects. However, it should be noted that the T0 screen guideline compliance of our intervention condition was significantly lower than our control condition. A regression to the mean effect may have influenced these results [77]. However, adjusting the model for screen time at T0 and conducting sensitivity analyses did not suggest that this phenomenon occurred. To the best of our knowledge, there are no intervention studies investigating effects on the use of screen time rules. However, instead of an intervention outcome variable, several interventions effectively encouraged setting up screen time rules as a strategy to reduce screen time in preschoolers and older children (0–18 years old) [78, 79]. In addition, the most frequently used intervention material in our intervention was the week schedule, with 45.5% of participants using it regularly. This tool included focussing on limiting screen time through planning and goal setting, which might have contributed to favourable intervention effects on screen rules and screen time guideline compliance. This is line with previous intervention studies in preschoolers that suggest that interventions using planning and goal setting strategies are effective in reducing screen time [23, 78]. With regard to the effect on explaining the rationale behind screen time rules, this can also be viewed as a parenting practice that fosters warmth and supports relatedness. According to research on children’s understanding of rules, preschoolers are more likely to internalize and comply with rules when they understand their purpose [80]. Furthermore, parental explanations contribute to children’s social and moral development, reinforcing their ability to recognize rules as a form of care and guidance rather than arbitrary restrictions [81, 82]. Overall, the effects we observed regarding screen time may be related to the importance parents attach to limiting it. The rapid digital evolution is accompanied by growing awareness of the potential negative effects of excessive screen time on cognitive, emotional, and physical development [83–86]. This may make parents more receptive to screen-related guidance and more motivated to reduce their child’s screen exposure.

Unexpectedly, the intervention had an unfavourable effect on physical activity guideline compliance, with the control condition having a significantly larger increase in compliance than the intervention condition. This is in contrast with other family-based intervention studies that generally show a favourable effect or no effect on physical activity in the early years [87]. It is unclear why this pattern occurred in our study. However, our results are likely due to model instability, possibly caused by quasi-complete separation, as nearly all participants in the control group were compliant with the PA guideline at T1. This is further supported by the wide confidence intervals observed. Nonetheless, there was a favourable effect on the parenting practice of parent and preschooler performing physical activity together. The review of Loprinzi and Trost (2010) emphasizes the favourable relationship between parental co-participation in physical activity and more physical activity in preschoolers [88]. Within the framework of the Self-Determination Theory, this co-participation can be seen as a parenting practice that fosters warmth and supports the basic psychological need of relatedness, rather than merely shared time together [24]. In our study, time spent on individual physical activity behaviour of the preschooler might have been replaced by physical activity together, leading to no effect on preschooler’s total physical activity, but could not be confirmed with our data. Nonetheless, by fulfilling the psychological need of relatedness, preschoolers might be motivated to perform physical activity in the long-term, which might be observable in a longitudinal study of a several years.

No intervention effect was found for complying with the sleep guideline. Intervention studies that did show favourable effects on sleep duration in the early years were predominantly sleep-only studies, whereas multi-component interventions were less beneficial [13], which might partly explain our result. However, previous sleep intervention studies used sleep diaries as measurement, which might have led to an overestimation of sleep [13]. Therefore, we should be careful when comparing results to our accelerometer based sleep data. In the evaluation of the implementation of our intervention, the session about sleep routine was perceived as the least novel content, with only 11.4% of participants considering the material as novel. Many parents indicated that they already followed a sleep routine, which might explain the lack of intervention effect on sleep guideline compliance. This finding is unexpected in light of the co-created development and prompts consideration of potential issues encountered during the co-creation process. It might be for example that focussing on a sleep routine was not really what parents needed or that the method was not applicable to focus on sleep routine. With regard to parenting practices, a favourable intervention effect was observed for providing choice within the sleep routine of the preschooler, with a similar medium effect size for both ITT and PP. Providing choice could, for example, be allowing the preschool child to select a bedtime story or which pyjama to wear. To our knowledge, there are no studies investigating the effect of an intervention on choice or autonomy support to improve sleep. The study of Joussemet et al. (2005) showed that 5 year old children with autonomy supportive parents, including providing choice, have better emotional and social adjustment three years later [89]. Extending this to sleep, providing choice might help to counter resistance when the preschool child does not want to go to bed as the child might emotionally better adjust to the fact that he/she has to go to bed. In the long term, the child might be more intrinsically motivated to go to bed in time [39]. No significant intervention effects from T0 to T1 were observed for the other parenting practices. One possible explanation is that participants already had high favourable scores for certain parenting practices (e.g. sleep routine) at T0, leaving little room for improvement.

The overall intervention effects on 24-hour movement behaviours and parenting practices were modest, with effects observed on a limited number of outcome variables. This could be due to low implementation rates, with parents attending only half (49.7%) of the sessions on average. Poor attendance has also been reported in previous family-based multicomponent interventions to improve health behaviours in preschoolers [23, 90]. For example, the study of Feng et al. (2024) also found that only about half of the parents attended the workshops, in their case online [23]. A possible explanation for these poor attendance rates could be busy time schedules and timing constraints in families with young children [23, 90]. This challenge was already taken into account during the co-creation sessions. As a result, the locations and time slots were selected based on what was most feasible for the majority of participants. Additionally, participants were welcome to join other groups at different locations and times if that better suited their availability for specific sessions throughout the intervention. Although this flexibility was offered, no participants opted to attend sessions with other groups. Despite these efforts, attendance rates remained lower than desired. Additionally, parents with valid data on T0 but invalid or no data at T1 were more likely to be part of the intervention condition than part of the control condition compared to participants with valid data on both timepoints. Parents likely perceived little value in completing T1 measurements if they were unable to attend the sessions, which further complicated evaluation efforts.

### Practical implications

Firstly, extended follow-up observations might be required to capture any meaningful effects on preschoolers’ 24-hour movement behaviours. The 11-week intervention period might be too short to observe significant changes in preschoolers’ 24-hour movement behaviours, especially given that the intervention targets preschoolers indirectly by first aiming to influence parents’ parenting practices. Since changes in parenting practices are a prerequisite for shifts in preschoolers’ 24-hour movement behaviours, a longer timeframe might be necessary. Secondly, to gain deeper insight into the mechanisms of the intervention, future studies are encouraged to conduct mediation analyses examining the role of parenting practices in behavioural change. Due to concerns about the robustness of certain models, such analyses were not included in the current study. Thirdly, future research should explore whether an intervention targeting all 24-hour movement behaviours simultaneously is the most effective strategy for improving these behaviours. While it is crucial for parents to understand the interrelated nature of these behaviours, our findings suggest that parents might prioritize some behaviours over others, particularly screen time. A more effective and feasible approach might be to support parents in focusing on their priority behaviour, rather than overwhelming them with intervention components targeting behaviours they do not intend to change (yet) [91]. These components for other behaviours might even be distracting and dilute the intervention impact. Focusing on adaptive intervention designs—where the type and dose of the intervention are tailored to the participant’s characteristics or progress, and repeatedly adjusted over time in response to changes in their behaviour—is considered a promising approach for achieving sustained intervention impact [92]. This tailoring could also involve exploring how intervention components might be embedded into activities that parents already value, such as integrating them into existing family habits and routines. This has been suggested as a promising strategy to foster parental engagement [93, 94]. For example, a family may value their traditional Friday pizza night. While waiting for the delivery, they could establish a new routine by playing an active game together, thereby incorporating physical activity into a moment they already enjoy. Fourthly, providing more concrete guidance on which parenting practices support or undermine the psychological needs of SDT for preschoolers may be beneficial in future interventions. For example, Ahmadi et al. (2023) developed a classification system of teacher motivational behaviours based on SDT to enhance students’ motivation, engagement, and learning [95]. A similar classification system could be useful for parents seeking to improve their practices to optimise their child’s behaviour, for practitioners supporting parents, and for researchers aiming to identify which parenting practices best predict child outcomes. Fifthly, strategies to improve parental attendance need further exploration. One possible solution is integrating intervention components into settings that parents already frequently visit, thereby reducing additional time commitments. For example, offering sessions immediately after school hours or in partnership with childcare services could increase accessibility. Previous studies suggest that interventions embedded within existing community programs yield higher engagement rates [96]. Additionally, hybrid models, including both in-person and online sessions, could help parents with varying schedules and availability [97, 98]. Nonetheless, each of these setting has its limitations and might only engage a select group of parents. To reach a broader and more diverse group, it might be necessary to implement the intervention across multiple settings. It may also be beneficial to allow more time during a co-creation process to explore potential barriers to attendance and accessibility of an intervention. These aspects may not have received sufficient attention during the co-creation of this intervention. Sixthly, to improve participant retention at T1, efforts should focus on increasing participant engagement. One solution might be to send reminders [99]. However, a reminder approach was already implemented in the current data collection and appeared to have limited effectiveness. As such, an email reminder was sent every five days, with a total of three reminders. Exploring personalized engagement strategies, such as emphasizing the importance of their contribution to the research or providing summary feedback on their child’s progress, may also help maintain parental involvement at T1 [100].

### Strengths and limitations

Strengths of the current study include (1) the combination of co-creation and IMP for intervention development, tailoring the intervention to the needs and preferences of parents; (2) applying an integrated approach taking into account the interrelationship between the 24-hour movement behaviours; (3) the objective measurement of 24-hour movement behaviours using accelerometers; (4) the inclusion of the secondary outcomes on parenting practices, which the intervention directly targeted as a means to promote more physical activity, less sedentary behaviour and a sufficient amount of sleep in preschoolers. Including this can provide insights into the mechanisms driving change in preschoolers’ 24-hour movement behaviours; (5) performing both ITT and PP analyses to study the “real-world” intervention effect and the intervention efficacy under more ideal conditions, which leads to more transparency of the findings [101].

This study also has some limitations: (1) the uneven distribution of participants across control and intervention groups might raise concerns about the robustness of the results. Uneven group sizes may introduce bias and lower statistical power when evaluating changes over time in intervention studies [102]; (2) the sample size calculation did not account for clustering at the preschool level, as the intervention was designed for implementation at the family level rather than being school-based. This may have reduced the statistical power of the models.; (3) this study had a quasi-experimental design and was no randomised-controlled trial, due to practical considerations as preschools were used for recruitment. This could have caused a selection bias; (4) Most parents were highly educated, which limits the generalizability of the results; (5) despite an initial focus on recruiting preschools with a high proportion of low-SEP families, response rates from these preschools were insufficient. Future research should explore alternative recruitment strategies to better reach low-SEP families, such as collaborating with community organizations or using trusted networks within low-SEP communities to enhance participation [103]; (6) most participating parents were mothers (71.6% in the control group and 86.4% in the intervention group), whereas fathers were welcome as well. More effort should be made to involve fathers as fathers’ involvement has been shown to provide unique benefits for preschoolers’ health behaviours, with research linking higher physical activity levels in preschoolers to an engaged and active father [104, 105].

## Conclusions

While the intervention led to some improvements in screen time guideline compliance and parents’ parenting practices related to screen time, physical activity and sleep, it did not significantly impact the 24-hour movement behaviour composition or overall guideline compliance. These null findings suggest that that interventions targeting multiple behaviours simultaneously may face challenges in achieving broad behavioural change within a limited timeframe. Extended follow-up observations might be required to capture changes in preschoolers’ 24-hour movement behaviours, especially considering that the intervention targets preschoolers indirectly by first aiming to influence parents’ parenting practices. The results furthermore suggest that planning and goal setting in a week schedule and through setting rules might be effective to reduce screen time. However, the lack of favourable effects on physical activity and sleep guideline compliance suggests that parents prioritise changing screen time over others. Future research should explore whether it would be more effective to support parents in addressing the behaviours they prioritise (i.e. screen time), rather than diverting their attention with intervention components targeting other behaviours. Moreover, low attendance rates and implementation challenges indicate the need for strategies to enhance parental interaction with the intervention, such as integrating intervention components into existing community settings or offering flexible delivery formats. Additionally, efforts should be made to engage diverse populations, particularly low-SEP families and fathers. Despite its limitations, this study underscores the potential of family-based approaches to change parents’ parenting practices focussing on preschoolers’ 24-hour movement behaviour and provides valuable insights for the development of future interventions. 

## Supplementary Information

Supplementary Material 1.

## Data Availability

The datasets analysed during the current study are available from the corresponding author on reasonable request.

## Abbreviations

BMI: Body Mass Index
CI: confidence interval
CoDA: compositional data analysis
ILR: isometric log-ratio
IMP: Intervention Mapping Protocol
ITT: intention-to-treat
LPA: light physical activity
MVPA: moderate-to-vigorous physical activity
OR: odds ratio
PP: per-protocol
SEP: socio-economic position
T0: pretest
T1: post-test
T2: follow-up test
WHO: World Health Organization
