# Supplementary material for: Optimising 24-Hour movement behaviours in preschoolers through parenting practices: an evidence-based intervention study
Source: Int J Behav Nutr Phys Act. 2025 Dec 12;23:4. doi: 10.1186/s12966-025-01863-z (PMC12821932; doi:10.1186/s12966-025-01863-z)
Supplement: Supplementary file 1 — Supplementary Material 1. [file 12966_2025_1863_MOESM1_ESM.docx]

**SUPPLEMENTARY FILE**

1. **CONSORT checklist and TIDieR checklist**

**A1. CONSORT checklist**

**
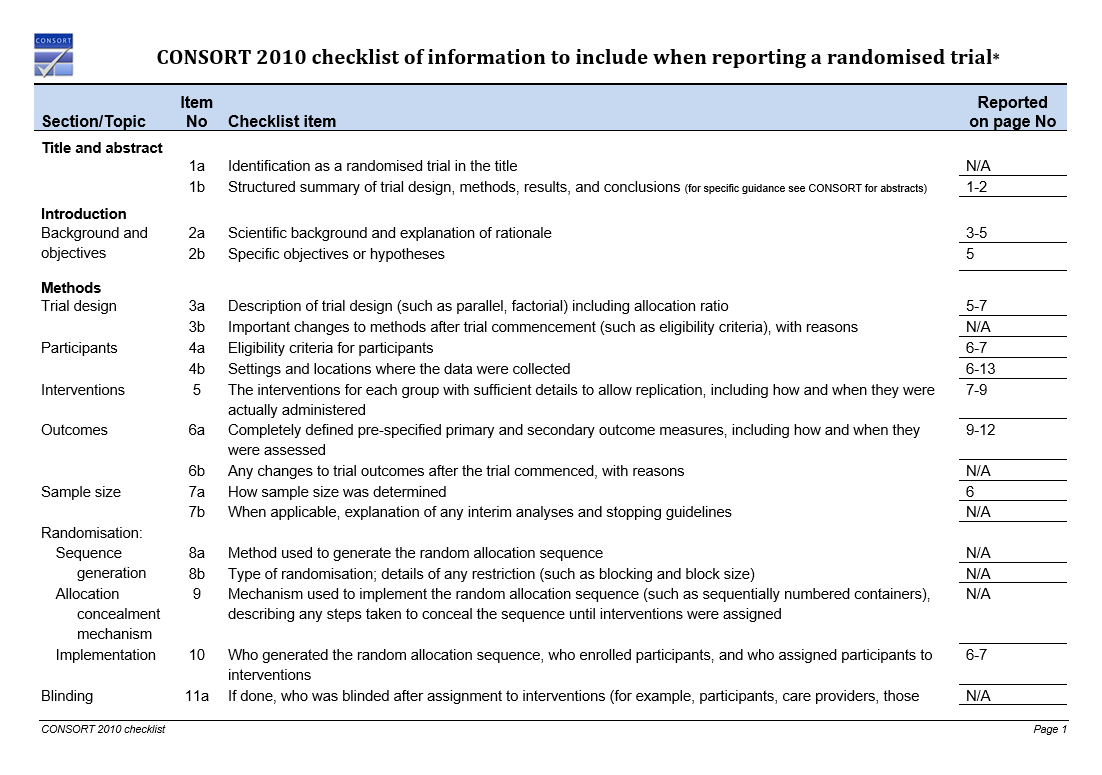
**

**
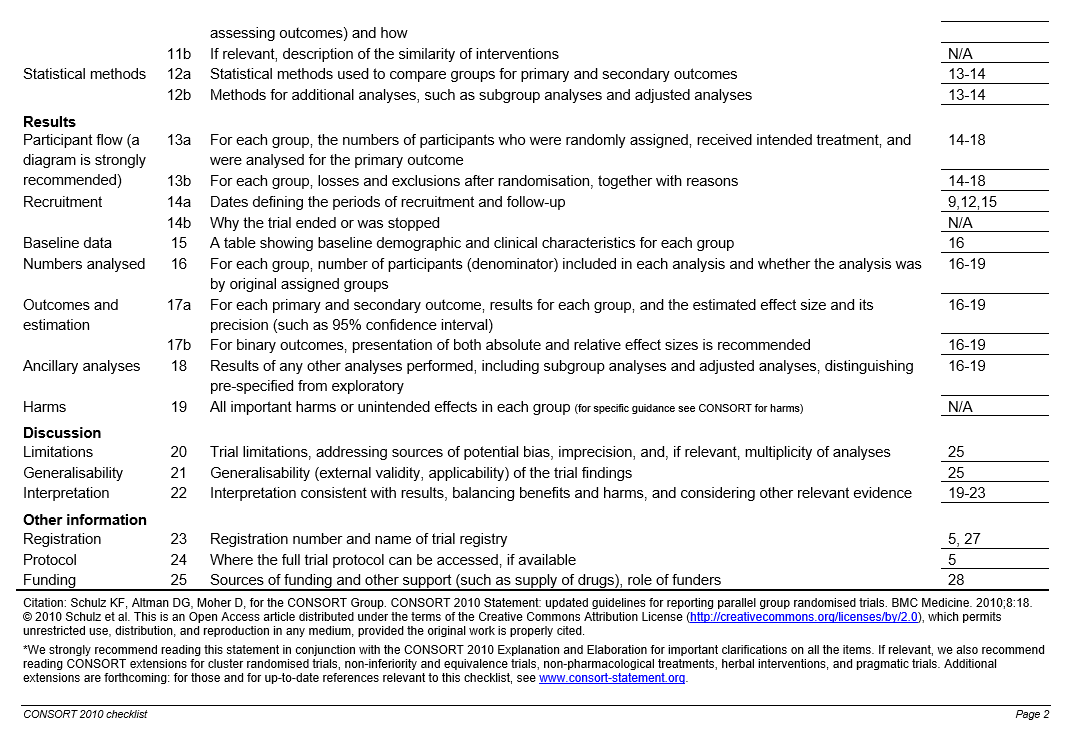
**

**A2. TIDieR checklist**

**
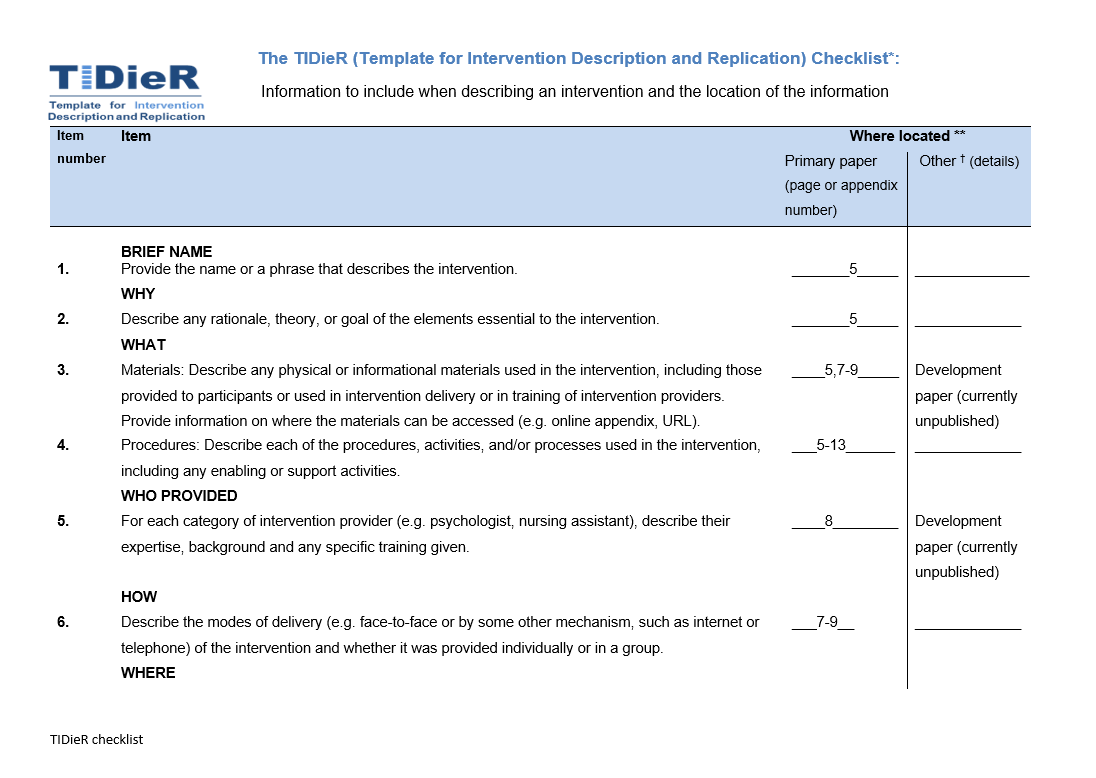
**

**
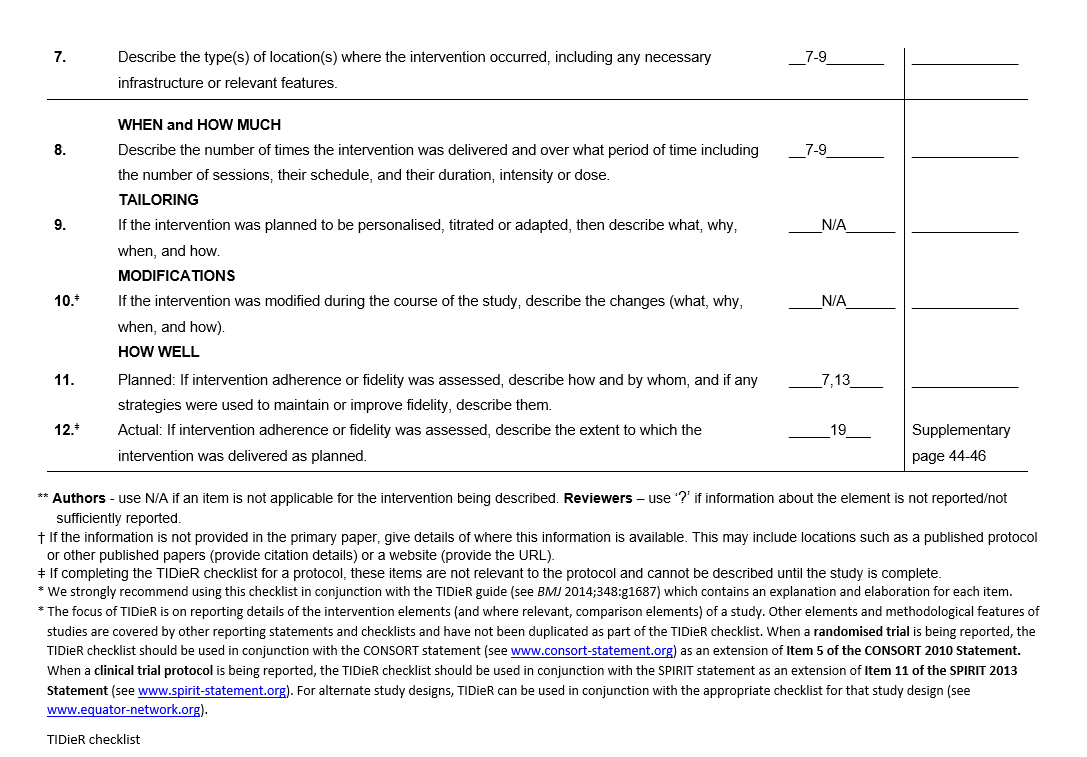
**

1. **Invalid and missing data analysis**

***Table B1 : Odds for valid data at the pretest (T0) but invalid or missing data at the post-test (T1) or follow-up (T2)***

| R1: n= 99 R2: n= 90 R3: n= 124 |  | Pre-post dropout  OR, 95%CI [lower;upper] | Pre-follow up dropout  OR, 95%CI [lower;upper] |
| --- | --- | --- | --- |
| Group (ref.:control) | R1 | 0.77 [0.20; 2.41] | NA |
|  | R2 | 1.26 [0.43; 3.47] | NA |
|  | R3 | **4.04 [1.59; 10.97]** | **3.98 [1.82; 8.96]** |
| Age child (in years) | R1 | 0.65 [0.33; 1.20] | NA |
|  | R2 | 0.77 [0.43; 1.34] | NA |
|  | R3 | 0.79 [0.45; 1.31] | **0.54 [0.33; 0.85]** |
| Age parent (in years) | R1 | 1.02 [0.90; 1.14] | NA |
|  | R2 | 0.97 [0.86; 1.09] | NA |
|  | R3 | 0.97 [0.87; 1.06] | 1.05 [0.97; 1.14] |
| Sex child (ref: male) | R1 | 0.84 [0.22; 2.39] | NA |
|  | R2 | 0.64 [0.23; 1.67] | NA |
|  | R3 | 1.34 [0.53; 3.35] | 1.03 [0.48; 2.21] |
| Sex parent (ref: male) | R1 | 0.69 [0.22; 2.39] | NA |
|  | R2 | 1.09 [0.37; 3.69] | NA |
|  | R3 | 1.00 [0.35; 3.30] | 1.47 [0.59; 4.08] |
| BMI z-score | R1 | 1.09 [0.61; 1.99] | NA |
|  | R2 | 1.05 [0.57; 1.89] | NA |
|  | R3 | 1.22 [0.71; 2.13] | 1.26 [0.80; 2.02] |
| BMI parent | R1 | 1.08 [0.94; 1.23] | NA |
|  | R2 | 1.03 [0.90; 1.16] | NA |
|  | R3 | 1.06 [0.98; 1.16] | 1.07 [0.99; 1.17] |
| Parental education level (ref: lower education) | R1 | 3.02e+07 [3.18e-51; NA]* | NA |
|  | R2 | 2.91[0.50; 55.21] | NA |
|  | R3 | **0.25 [0.08; 0.78]** | 0.36 [0.13; 1.03] |

R1=intervention-effect on 24-hour movement behaviour composition , R2=intervention-effect on 24-hour movement behaviour guidelines ,R3=intervention-effect on parenting practices, OR = odds ratio, 95%CI = 95% confidence interval, ref.=reference category, BMI=Body Mass Index.
*Result due to small group with lower educational level (n=24), no one with lower educational level had valid data on T0 but invalid or missing data at T1.

p<0.05

1. **Descriptive characteristics of participants – sample for the secondary outcomes**

***Table C1: Descriptive characteristics of participants – sample for the secondary outcomes***

|  | control | ITT | PP | p-value (C-ITT) | p-value (C-PP) |
| --- | --- | --- | --- | --- | --- |
| N | 77 | 44 | 22 |  |  |
| Age child in years (mean (SD)) | 4.16 (0.91) | 3.93 (0.88) | 4.23 (0.90) | 0.17 | 0.74 |
| Age parent in years (mean (SD)) | 36.61 (5.04) | 35.85 (4.71) | 36.64 (3.68) | 0.41 | 0.98 |
| Sex child = female (%) | 33 (42.9) | 19 (40.4) | 12 (54.5) | 0.94 | 0.47 |
| Sex parent = female (%) | 56 (72.7) | 41 (87.2) | 18 (81.8) | 0.09 | 0.56 |
| BMI z-score child (mean (SD)) | **0.34 (0.80)** | **0.70 (0.87)** | 0.55 (1.03) | **0.02** | 0.30 |
| BMI parent (mean (SD)) | 23.64 (3.65) | 24.56 (6.71) | 23.53 (4.51) | 0.32 | 0.91 |
| Parental education level = higher (%) | 69 (89.6) | 38 (80.9) | 19 (86.4) | 0.27 | 0.97 |

N=number, SD=standard deviation, BMI = Body Mass Index, ITT=intention-to-treat, PP=per-protocol

1. **Variance for clustering School**

***Table D1: Means, interaction effects and changes in parenting practices from pretest to post-test and follow-up between intervention and control group***

| Parenting practices | Group comparison | Variance of random-effect school (SD) | Post-hoc ICC esimate |
| --- | --- | --- | --- |
| PA choice | ITT-C | 0.001 (0.04) | <0.01 |
|  | PP-C | 0.001 (0.04) | <0.01 |
| ST alternatives | ITT-C | 0.007 (0.09) | 0.01 |
|  | PP-C | 0.006 (0.08) | 0.01 |
| choice in sleep routine | ITT-C | 0.00 (0.00) | 0.00 |
|  | PP-C | 8.6e-11 (9.3e-06) | <0.01 |
| PA compliment | ITT-C | 0.08 (0.28) | 0.10 |
|  | PP-C | 0.11 (0.34) | 0.14 |
| PA together | ITT-C | 0.02 (0.15) | 0.04 |
|  | PP-C | 0.03 (0.16) | 0.05 |
| ST rules explanation | ITT-C | 0.15 ( 0.38) | 0.09 |
|  | PP-C | 0.15 (0.38) | 0.09 |
| PA plan | ITT-C | 0.14 (0.37) | 0.10 |
|  | PP-C | 0.14 (0.38) | 0.11 |
| ST rules | ITT-C | 0.12 (0.34) | 0.08 |
|  | PP-C | 0.12 (0.35) | 0.09 |
| monitor ST | ITT-C | 0.04 (0.20) | 0.03 |
|  | PP-C | 0.04 (0.21) | 0.03 |
| fixed bedtime | ITT-C | 0.04 (0.20) | 0.09 |
|  | PP-C | 0.03 (0.19) | 0.08 |
| sleep routine | ITT-C | 4.1e-11 (6.46e-06) | <0.01 |
|  | PP-C | 0.00 (0.00) | 0.00 |

1. **Intervention-effects for the secondary outcomes, parenting practices, at follow-up (T2)**

***Table E1: Means, interaction effects and changes in parenting practices from pretest to post-test and follow-up between intervention and control group***

| **Outcomes** | **Group** | **Descriptive statistics** | | | **Mixed model results** | | | | |
| --- | --- | --- | --- | --- | --- | --- | --- | --- | --- |
|  |  | Pretest | Post-test | Follow-up | Adjusted mean difference (intervention vs. control)  Estimate (SE) [95%CI] | | | Time x Group p-value (effect size) | |
| N | ITT | 44 | 29 | 23 |  |  |  |  |  |
|  | PP | 22 | 21 | 20 |  |  |  |  |  |
|  | C | 77 | 71 | 63 |  |  |  |  |  |
|  | | Adjusted mean (SE) | | |  | Pre-post | Pre-follow-up | Pre-post | Pre-follow-up |
| **Autonomy support** | | | | | | | | | |
| PA choice | ITT | 3.91 (0.11) | 3.97 (0.13) | 4.26 (0.14) | ITT-C | -0.01 (0.16) [-0.31; 0.30] | **0.39 (0.17) [0.06; 0.73]** | 0.97 (-01) | **0.02 (0.76)** |
|  | PP | 3.95 (0.15) | 3.95 (0.16) | 4.25 (0.16) | PP-C | -0.08 (0.17) [-0.42; 0.27] | **0.33 (0.18) [-0.01; 0.69]** | 0.66 (-0.14) | 0.06 (0.65) |
|  | C | 4.07 (0.09) | 4.14 (0.10) | 4.03 (0.10) |  |  |  |  |  |
| ST alternatives | ITT | 4.14 (0.11) | 4.35 (0.14) | 4.10 (0.15) | ITT-C | 0.19 (0.19) [-0.18; 0.56] | 0.08 (0.20) [-0.32; 0.48] | 0.32 (0.29) | 0.69 (0.13) |
|  | PP | 4.23 (0.16) | 4.48 (0.16) | 4.14 (0.17) | PP-C | 0.23 (0.22) [-0.20; 0.67] | 0.04 (0.22) [-0.40; 0.48] | 0.29 (0.37) | 0.86 (0.06) |
|  | C | 4.29 (0.10) | 4.32 (0.10) | 4.17 (0.11) |  |  |  |  |  |
| choice in sleep routine | ITT | 4.11 (0.12) | 4.46 (0.15) | 4.24 (0.16) | ITT-C | **0.41 (0.20) [0.03; 0.80]** | 0.15 (0.21) [-0.26; 0.56] | **0.04 (0.63)** | 0.48 (0.23) |
|  | PP | 4.18 (0.17) | 4.57 (0.18) | 4.27 (0.18) | PP-C | **0.45 (0.23) [-0.00; 0.90]** | 0.12 (0.23) [-0.35; 0.58] | **0.05 (0.68)** | 0.62 (0.17) |
|  | C | 4.30 (0.11) | 4.24 (0.11) | 4.27 (0.12) |  |  |  |  |  |
| **Warmth** | | | | | | | | | |
| PA compliment | ITT | 3.85 (0.15) | 4.19 (0.18) | 3.97 (0.19) | ITT-C | 0.13 (0.19) [ -0.25; 0.51] | -0.12 (0.21) [-0.53; 0.29] | 0.50 (0.20) | 0.56 (-0.19) |
|  | PP | 4.22 (0.21) | 4.37 (0.21) | 4.17 (0.22) | PP-C | -0.06 (0.22) [-0.50; 0.38] | -0.29 (0.23) [-0.74; 0.16] | 0.77 (-0.10) | 0.21 (-0.46) |
|  | C | 3.80 (0.14) | 4.02 (0.14) | 4.05 (0.14) |  |  |  |  |  |
| PA together | ITT | 3.29 (0.12) | 3.65 (0.14) | 3.39 (0.15) | ITT-C | 0.28 (0.164) [-0.04; 0.60] | 0.14 (0.18) [-0.20; 0.49] | 0.09 (0.52) | 0.42 (0.26) |
|  | PP | 3.31 (0.17) | 3.81 (0.17) | 3.51 (0.17) | PP-C | **0.41 (0.19) [0.04; 0.79]** | 0.24 (0.19) [-0.14; 0.62] | **0.03 (0.76)** | 0.21 (0.45) |
|  | C | 3.52 (0.11) | 3.60 (0.11) | 3.47 (0.11) |  |  |  |  |  |
| ST rules explanation | ITT | 3.36 (0.22) | 3.65 (0.24) | 3.58 (0.26) | ITT-C | 0.36 (0.229) [-0.0868 0.815] | 0.21 (0.247) [-0.28; 0.69 ] | 0.11 (0.49) | 0.41 (0.27) |
|  | PP | 3.42 (0.30) | 3.85 (0.30) | 3.72 (0.30) | PP-C | 0.51 (0.27) [-0.01; 1.04] | 0.29 (0.27) [-0.24; 0.83] | 0.05 (0.68) | 0.28 (0.39) |
|  | C | 3.43 (0.20) | 3.35 (0.20) | 3.44 (0.20) |  |  |  |  |  |
| **Structure** | | | | | | | | | |
| PA plan | ITT | 3.29 (0.18) | 3.21 (0.18) | 3.54 (0.19) | ITT-C | -0.17 (0.26) [-0.69; 0.34] | 0.12 (0.28)[-0.44 0.67] | 0.51 (-0.20) | 0.68 (0.13) |
|  | PP | 3.24 (0.27) | 3.19 (0.27) | 3.49 (0.28) | PP-C | -0.14 (0.30) [-0.74; 0.46] | 0.12 (0.31) [-0.49; 0.73] | 0.65 (-0.16) | 0.70 (0.14) |
|  | C | 3.60 (0.18) | 3.69 (0.18) | 3.73 (0.19) |  |  |  |  |  |
| ST rules | ITT | 3.36 (0.20) | 3.99 (0.23) | 4.02 (0.24) | ITT-C | **0.63 (0.24) [0.15; 1.10]** | 0.47 (0.26) [-0.05; 0.98] | **0.01 (0.79)** | 0.08 (0.58) |
|  | PP | 3.10 (0.27) | 3.98 (0.27) | 3.96 (0.28) | PP-C | **0.89 (0.27) [0.35; 1.42]** | **0.67(0.28) [0.12; 1.22]** | **0.001 (1.1)** | **0.02 (0.84)** |
|  | C | 3.76 (0.18) | 3.76 (0.18) | 3.95 (0.18) |  |  |  |  |  |
| monitor ST | ITT | 2.82 (0.20) | 3.29 (0.23) | 3.32 (0.25) | ITT-C | 0.46 (0.25) [-0.03; 0.94] | 0.52 (0.27) [-0.01; 1.04] | 0.07 (0.56) | 0.05 (0.64) |
|  | PP | 2.92 (0.28) | 3.41 (0.28) | 3.49 (0.28) | PP-C | 0.48 (0.28) [-0.06; 1.03] | **0.59 (0.28) [0.03; 1.15]** | 0.08 (0.59) | **0.04 (0.72)** |
|  | C | 3.33 (0.17) | 3.34 (0.18) | 3.31 (0.18) |  |  |  |  |  |
| fixed bedtime | ITT | 4.30 (0.11) | 4.11 (0.13) | 4.21 (0.14) | ITT-C | -0.16 (0.15) [-0.46 0.14] | -0.27 (0.16) [-0.59; 0.05] | 0.30 (-0.31) | 0.10 (-0.54) |
|  | PP | 4.10 (0.15) | 3.96 (0.15) | 4.14 (0.16) | PP-C | -0.11 (0.18) [-0.46; 0.25] | -0.14 (0.18) [-0.50; 0.22] | 0.55 (-0.21) | 0.45 (-0.29) |
|  | C | 4.31 (0.10) | 4.28 (0.10) | 4.49 (0.10) |  |  |  |  |  |
| sleep routine | ITT | 4.68 (0.09) | 4.58 (0.10) | 4.76 (0.10) | ITT-C | -0.05 (0.09) [-0.22; 0.12] | 0.11 (0.09) [-0.07 0.30] | 0.57 (-0.17) | 0.23 (0.40) |
|  | PP | 4.68 (0.12) | 4.54 (0.12) | 4.76 (0.13) | PP-C | -0.09 (0.10) [-2.94e-01; 0.11] | 0.11 (0.10) [-9.13e-02; 0.32] | 0.36 (-0.33) | 0.27 (0.40) |
|  | C | 4.77 (0.08) | 4.72 (0.08) | 4.74 (0.08) |  |  |  |  |  |

ITT=Intervention intention-to-treat group; PP= intervention per-protocol group; C=Control group; ST=screen time; PA=physical activity; SE=standard error; T0 = pretest; T1 = post-test; T2=follow-up; CI=Confidence Interval

1. **Variation matrices**

***Table E1: Pair-wise log-ratio variation matrices for sleep, SB, LPA, MVPA in minutes/day***

|  | Sleep | SB | LPA | MVPA |
| --- | --- | --- | --- | --- |
|  | Control group T0 |  |  |  |
| Sleep | 0.000 | 0.048 | 0.046 | 0.129 |
| SB | 0.048 | 0.000 | 0.063 | 0.170 |
| LPA | 0.046 | 0.063 | 0.000 | 0.115 |
| MVPA | 0.129 | 0.170 | 0.115 | 0.000 |
|  | ITT group T0 |  |  |  |
| Sleep | 0.000 | 0.052 | 0.023 | 0.091 |
| SB | 0.052 | 0.000 | 0.048 | 0.137 |
| LPA | 0.023 | 0.048 | 0.000 | 0.068 |
| MVPA | 0.091 | 0.137 | 0.068 | 0.000 |
|  | PP group T0 |  |  |  |
| Sleep | 0.000 | 0.067 | 0.021 | 0.122 |
| SB | 0.067 | 0.000 | 0.051 | 0.174 |
| LPA | 0.021 | 0.051 | 0.000 | 0.091 |
| MVPA | 0.122 | 0.174 | 0.091 | 0.000 |
|  | Control group T1 |  |  |  |
| Sleep | 0.000 | 0.045 | 0.023 | 0.078 |
| SB | 0.045 | 0.000 | 0.044 | 0.142 |
| LPA | 0.023 | 0.044 | 0.000 | 0.086 |
| MVPA | 0.078 | 0.142 | 0.086 | 0.000 |
|  | ITT group T1 |  |  |  |
| Sleep | 0.000 | 0.056 | 0.020 | 0.106 |
| SB | 0.056 | 0.000 | 0.044 | 0.212 |
| LPA | 0.020 | 0.044 | 0.000 | 0.120 |
| MVPA | 0.106 | 0.212 | 0.120 | 0.000 |
|  | PP group T1 |  |  |  |
| Sleep | 0.000 | 0.057 | 0.020 | 0.125 |
| SB | 0.057 | 0.000 | 0.031 | 0.227 |
| LPA | 0.020 | 0.031 | 0.000 | 0.141 |
| MVPA | 0.125 | 0.227 | 0.141 | 0.000 |

SB=sedentary behaviour, LPA=light physical activity, MVPA=moderate-to-vigorous physical activity, T0=pretest, T1=post-test

1. **Indication for regression to the mean**

Based on the descriptive statistics of complying with the ST guideline (see main paper), there might be an indication for a regression to the mean effect instead of an intervention-effect on the ST guideline compliance. A suggested way to check for this regression to the mean effect is controlling for the baseline (T0) measurement (1). In table F1 the ST variable at T0 is added as a covariate in the analyses (see results for ST2). In addition, to provide a broader view on the data, some sensitivity analyses were added (see results for ST3 and ST4)

***Table G1: Additional analyses for the intervention-effect on complying to the ST guideline to provide insight in a potential regression to the mean effect***

| **Outcomes** | **Group** | **% guideline compliance** | | **Mixed model results T1-T0** | | |
| --- | --- | --- | --- | --- | --- | --- |
|  |  | T0 | T1 | Time x Group  Ref. = control group and pretest | | |
|  |  |  |  | Groups | OR [95%CI] | p-value |
| ST1 | ITT | 29.2 | 77.8 | C-ITT | **14.7 [1.2, 179.6]** | **0.04** |
|  | PP | 31.6 | 80.0 | C-PP | **5.7e+06 [7.8e+03, 4.2e+09]** | **<0.001** |
|  | C | 62.1 | 69.6 |  |  |  |
| ST2 | ITT | 29.2 | 77.8 | C-ITT | **23.3 [2.4, 228.4]** | **0.007** |
|  | PP | 31.6 | 80.0 | C-PP | **48.6 [3.5, 667.2]** | **0.004** |
|  | C | 62.1 | 69.6 |  |  |  |
| ST3 | ITT | 34.8 | 81.2 | C-ITT | **21.1 [1.4, 305.1]** | **0.03** |
|  | PP | 35.3 | 91.7 | C-PP | **1.3e+07 [2.5e+03, 6.5e+10]** | **<0.001** |
|  | C | 62.1 | 69.6 |  |  |  |
| ST4 | ITT | 38.1 | 72.2 | C-ITT | 9.2 [0.81, 103.9] | 0.07 |
|  | PP | 37.5 | 78.6 | C-PP | **2.8e+06 [3.2e+03, 2.4e+09]** | **<0.001** |
|  | C | 62.1 | 69.6 |  |  |  |

ST=screen time; ITT=Intervention intention-to-treat group; PP= intervention per-protocol group; C=Control group;T0 = pretest; T1 = post-test; OR=Odds Ratio; CI=Confidence Interval; Ref.=reference category;ST1=Results for sample in the main paper; ST2=Results when including covariate ST at T0; ST3= Results for sample excluding 20% of participants in the intervention group (n=4) with the highest ST on baseline T0; ST4: Results for sample excluding participants of the intervention group just not meeting the ST guideline on baseline T0. Their ST is 60 to 65 minutes. Based on this criterium 4 participants were excluded.

1. **Implementation analyses**

Table H1 represents each implementation item that was used to create an implementation score. Each item was recoded into a binary item (0 and 1), where 1 indicated a higher level of implementation. Per item the table presents the number and percentage of participants that scored 1 per session or per material. Also, the adjusted mean and median is presented per item. This is based on the sum score of all subparts of that item, e.g. the scores for all sessions are summed and then recoded on a scale of 10. The mean of the score on 10 is the adjusted mean. For each intervention part (e.g., sessions delivered, materials delivered), a sum score was created and rescaled to a score on 10 to weigh each part equally. Subsequently, a sum score was created for dose delivered, dose received - exposure, and – satisfaction separately and rescaled to 10. These three components were then summed to produce a total implementation score, where higher values indicated better implementation.

***Table H1: Implementation of the intervention: dose delivered and dose received (n=44)***

| **Dose delivered = how much was delivered by the researchers** | | | |
| --- | --- | --- | --- |
| *(L) Session delivered (based on presence of participants)*  **1 = yes n(%):**   - Session 1: 29 (65.9) - Session 2: 27 (61.4) - Session 3: 21 (47.7) - Session 4: 19 (43.2) - Session 5: 19 (43.2) - Session 6: 19 (43.2) - Session 7: 19 (43.2)   **0 = no**  Adj. mean (SD): 4.97 (3.84)/10  Adj. median (1q, 3q): 5.00  (1.43, 8.57)/10 | | *(L) Material delivered*  **1 = yes n(%):**   - PPT S1: 36 (81.8) - Game pot S1: 35 (79.5) - Calendar S1: 35 (79.5) - Week schedule S2: 36 (81.8) - Parent tips S3: 21 (47.7) - Movement games S4: 32 (72.7) - Sleep routine S5: 27 (61.4) - Parent tips S6: 19 (43.2) - Sustainability tips S7: 19 (43.2) - Materialbox^Δ^: 20 (45.5)   **0 = no**  Adj. mean (SD): 5.85 (3.89)/10  Adj. median (1q, 3q): 7.50 (2.50, 10.00)/10 | |
| Total dose delivered: Adj. mean (SD): 5.41 (3.83)/10  Adj. median (1q, 3q): 6.25 (1.96, 8.82)/10 | | | |
| **Dose received – exposure = was the intervention received by the participants** | | | |
| (SF) *Use of materials*  **1 = yes n(%):**   - Calendar: 21 (47.7) - Game pots: 18 (40.9) - Materialbox: 22 (50.0) - Week schedule: 26 (59.1) - Movement games: 21 (47.7) - Sleep routine: 17 (38.6)   **0 = no**  Adj. mean (SD): 4.73 (4.31)/10  Adj. median (1q, 3q): 5.00 (0.00, 10.00)/10 | | (SF) *Frequency of material use**  **1 = score 3,4 and 5 n(%):**   - Calendar: 6 (13.6) - Game pots: 9 (20.5) - Materialbox: 16 (36.4) - Week schedule: 20 (45.5) - Movement games: 10 (22.7) - Sleep routine: 16 (36.4)   **0 = score 1 and 2**  Adj. mean (SD): 2.92 (3.26)/10  Adj. median (1q, 3q): 1.67 (0.00, 5.00)/10 | |
| Total dose received-exposure: Adj. mean (SD): 3.83 (3.64)/10  Adj. median (1q, 3q): 3.33 (0.00, 6.88)/10 | | | |
| **Dose received – satisfaction = how was the intervention received by the participants** | | | |
| (SF) Was the content new per session?*  **1 = score 3,4 and 5** **n(%):**   - Session 1: 21 (47.7) - Session 2: 19 (43.2) - Session 3: 18 (40.9) - Session 4: 12 (27.3) - Session 5: 5 (11.4) - Session 6: 16 (36.4) - Session 7: 14 (31.8)   **0 = score 1 and 2**  Adj. mean (SD): 3.41 (3.29)/10  Adj. median (1q, 3q): 2.86 (0.00, 7.14)/10 | (SF) Did you believe in the content per session?*  **1 = score 3,4 and 5 n(%):**   - Session 1: 28 (63.6) - Session 2: 25 (56.8) - Session 3: 20 (45.5) - Session 4: 18 (40.9) - Session 5: 18 (40.9) - Session 6: 19 (43.2) - Session 7: 17 (38.6)   **0 = score 1 and 2**  Adj. mean (SD): 4.74 (3.87)/10  Adj. median (1q, 3q): 4.29 (1.07, 8.57)/10 | | (SF) Do you think you can apply the content at home per session?*  **1 = score 3,4 and 5 n(%):**   - Session 1: 28 (63.6) - Session 2: 25 (56.8) - Session 3: 19 (43.2) - Session 4: 18 (40.9) - Session 5: 18 (40.9) - Session 6: 19 (43.2) - Session 7: 17 (38.6)   **0 = score 1 and 2**  Adj. mean (SD): 4.71 (3.84)/10  Adj. median (1q, 3q): 4.29 (1.07, 8.57)/10 |
| (SF) Was the goal clear per session?*  **1 = score 3,4 and 5 n(%):**   - Session 1: 28 (63.6) - Session 2: 25 (56.8) - Session 3: 21 (47.7) - Session 4: 16 (36.4) - Session 5: 18 (40.9) - Session 6: 18 (40.9) - Session 7: 17 (38.6)   **0 = score 1 and 2**  Adj. mean (SD): 4.64 (3.82)/10  Adj. median (1q, 3q): 4.29 (1.07, 8.57)/10 | (SF) Were you satisfied per session?*  **1 = score 3,4 and 5 n(%):**   - Session 1: 28 (63.6) - Session 2: 26 (59.1) - Session 3: 21 (47.7) - Session 4: 17 (38.6) - Session 5: 18 (40.9) - Session 6: 19 (43.2) - Session 7: 17 (38.6)   **0 = score 1 and 2**  Adj. mean (SD): 4.77 (3.90)/10  Adj. median (1q, 3q): 4.29 (1.07, 8.57)/10 | | (SF) Are you motivated to continue working with what was provided per session?*  **1 = score 3,4 and 5 n(%):**   - Session 1: 28 (63.6) - Session 2: 26 (59.1) - Session 3: 17 (38.6) - Session 4: 18 (40.9) - Session 5: 18 (40.9) - Session 6: 16 (36.4) - Session 7: 17 (38.6)   **0 = score 1 and 2**  Adj. mean (SD): 4.55 (3.64)/10  Adj. median (1q, 3q): 4.29 (1.07, 8.57)/10 |
| (PPE) What was your overall impression of the program?  **1= I liked it a lot (4), I liked it (3) n(%):** 27 (73.0)  **0 = I did not really like it (2), I did not like it at all (1), I don’t know that much about it (0)**  Adj. mean (SD): 6.14 (4.93)/10  Adj. median (1q, 3q): 10.00 (0.00, 10.00)/10 | (PPE) Did the project meet your expectations  **1 = neutral (2), somewhat agree (3), strongly agree (4) n(%):** 28 (75.7)  **0 = strongly disagree (0), somewhat disagree (1)**  Adj. mean (SD): 6.36 (4.87)/10  Adj. median (1q, 3q): 10.00 (0.00, 10.00)/10 | | (PPE) How do you rate the organization of the project?  **1 = neutral (2), rather good (3), very good (3) n(%):** 28 (75.7)  **0 = not at all good (0), rather not good (1)**  Adj. mean (SD): 6.36 (4.87)/10  Adj. median (1q, 3q): 10.00 (0.00, 10.00)/10 |
| (PPE) *Did the project motivate you to change something with regard to the behaviours?**  **1 = score 3,4 and 5 n(%):**   - physical activity: 24 (54.5) - screen time: 18 (40.9) - sleep: 7 (15.9)   **0 = score 1 and 2**  Adj. mean (SD): 3.71 (3.75)/10  Adj. median (1q, 3q): 3.33 (0.00, 6.67)/10 | (PPE) Which materials provided in the project do you want to use further? Per material:  **1 = yes n(%):**   - Calendar: 24 (54.5) - Game pots: 24 (54.5) - Week schedule: 17 (38.6) - Movement games: 28 (63.6) - Sleep routine: 26 (59.1) - Sustainable behaviour tips: 21 (47.7)   **0 = no**  Adj. mean (SD): 5.30 (3.79)/10  Adj. median (1q, 3q): 5.00 (1.67, 10.00)/10 | |  |
| Total dose received-satisfaction  Adj. mean (SD): 4.95 (3.89) /10  Adj. median (1q, 3q): 5.34 (0.69, 8.99)/10 | | | |
| Total implementation score:  Adj. mean (SD): 4.73 (3.67)/10  Adj. median (1q, 3q): 5.41 (0.91, 8.44)/10 | | | |

Adj=adjusted by rescaling to a score /10; SD=standard deviation; 1q,3q=1th and 3th quartile

*for these items participants could give a score from 1 to 5 with “1 = not at all” to “5 = to high degree”;

^Δ^ Materialbox was recoded into an average score to avoid too much weighing of this material. In 5 of the 7 sessions, children could use the box;

(L) items were collected via a logbook completed by the researchers;

(SF) items were collected via questionnaires at the end of each session;

(PPE) items were collected via a questionnaire at the post-test;

Note that missings were recoded into 0 for all implementation parts.

1. Barnett AG, van der Pols JC, Dobson AJ. Regression to the mean: what it is and how to deal with it. Int J Epidemiol. 2005;34(1):215-20.
